# Regulation of Gene Expression in Autoimmune Disease Loci and the Genetic Basis of Proliferation in CD4^+^ Effector Memory T Cells

**DOI:** 10.1371/journal.pgen.1004404

**Published:** 2014-06-26

**Authors:** Xinli Hu, Hyun Kim, Towfique Raj, Patrick J. Brennan, Gosia Trynka, Nikola Teslovich, Kamil Slowikowski, Wei-Min Chen, Suna Onengut, Clare Baecher-Allan, Philip L. De Jager, Stephen S. Rich, Barbara E. Stranger, Michael B. Brenner, Soumya Raychaudhuri

**Affiliations:** 1 Division of Rheumatology, Immunology and Allergy, Department of Medicine, Brigham and Women's Hospital, Boston, Massachusetts, United States of America; 2 Division of Genetics, Department of Medicine, Brigham and Women's Hospital, Boston, Massachusetts, United States of America; 3 Partners Center for Personalized Genetic Medicine, Boston, Massachusetts, United States of America; 4 Program in Medical and Population Genetics, Broad Institute of MIT and Harvard, Cambridge, Massachusetts, United States of America; 5 Harvard Medical School, Boston, Massachusetts, United States of America; 6 Harvard-MIT Division of Health Sciences and Technology, Boston, Massachusetts, United States of America; 7 Program in Translational NeuroPsychiatric Genomics, Institute for the Neurosciences, Department of Neurology, Brigham and Women's Hospital, Boston, Massachusetts, United States of America; 8 Center for Public Health Genomics, University of Virginia, Charlottesville, Virginia, United States of America; 9 Department of Dermatology/Harvard Skin Disease Research Center, Brigham and Women's Hospital, Boston, Massachusetts, United States of America; 10 Section of Genetic Medicine, University of Chicago, Chicago, Illinois, United States of America; 11 Institute for Genomics and Systems Biology, University of Chicago, Chicago, Illinois, United States of America; 12 Faculty of Medical and Human Sciences, University of Manchester, Manchester, United Kingdom; The Jackson Laboratory, United States of America

## Abstract

Genome-wide association studies (GWAS) and subsequent dense-genotyping of associated loci identified over a hundred single-nucleotide polymorphism (SNP) variants associated with the risk of rheumatoid arthritis (RA), type 1 diabetes (T1D), and celiac disease (CeD). Immunological and genetic studies suggest a role for CD4-positive effector memory T (CD^+^ T_EM_) cells in the pathogenesis of these diseases. To elucidate mechanisms of autoimmune disease alleles, we investigated molecular phenotypes in CD4^+^ effector memory T cells potentially affected by these variants. In a cohort of genotyped healthy individuals, we isolated high purity CD4^+^ T_EM_ cells from peripheral blood, then assayed relative abundance, proliferation upon T cell receptor (TCR) stimulation, and the transcription of 215 genes within disease loci before and after stimulation. We identified 46 genes regulated by *cis*-acting expression quantitative trait loci (eQTL), the majority of which we detected in stimulated cells. Eleven of the 46 genes with eQTLs were previously undetected in peripheral blood mononuclear cells. Of 96 risk alleles of RA, T1D, and/or CeD in densely genotyped loci, eleven overlapped *cis*-eQTLs, of which five alleles completely explained the respective signals. A non-coding variant, rs389862^A^, increased proliferative response (*p* = 4.75×10^−8^). In addition, baseline expression of seventeen genes in resting cells reliably predicted proliferative response after TCR stimulation. Strikingly, however, there was no evidence that risk alleles modulated CD4^+^ T_EM_ abundance or proliferation. Our study underscores the power of examining molecular phenotypes in relevant cells and conditions for understanding pathogenic mechanisms of disease variants.

## Author Summary

Genome-wide association studies have identified hundreds of genetic variants associated to autoimmune diseases. To understand the mechanisms and pathways affected by these variants, follow-up studies of molecular phenotypes and functions are required. Given the diversity of cell types and specialization of functions within the immune system, it is crucial that such studies focus on specific and relevant cell types. Here, we studied genetic and cellular traits of CD4-positive effector memory T (CD4^+^ T_EM_) cells, which are particularly important in the onset of rheumatoid arthritis, celiac disease, and type 1 diabetes. In a cohort of healthy individuals, we purified CD4^+^ T_EM_ cells, assayed genome-wide single nucleotide polymorphisms (SNPs), abundance of CD4^+^ T_EM_ cells in blood, proliferation upon T cell receptor stimulation, and 215 gene transcripts in resting and stimulated states. We found that expression levels of 46 genes were regulated by nearby SNPs, including disease-associated SNPs. Many of these expression quantitative trait loci were not previously seen in studies of more heterogeneous peripheral blood cells. We demonstrated that relative abundance and proliferative response of CD4^+^ T_EM_ cells varied in the population, however disease alleles are unlikely to confer risk by modulating these traits in this cell type.

## Introduction

Memory T cells are an important component of the adaptive immune system. They circulate between lymphoid organs, blood, and peripheral tissues, and facilitate faster and more aggressive immune response to antigens after re-exposure. CD4-positive effector memory T (CD4^+^ T_EM_) cells are known to migrate to peripheral sites of inflammation upon activation, and rapidly produce both Th1 and Th2 cytokines [1]. Investigators have long suggested their involvement in autoimmune diseases including rheumatoid arthritis (RA), type I diabetes (T1D), and celiac disease (CeD) [2]–[5]. However, whether changes in cell population subsets and functions are causal or reactive to disease is uncertain. One strategy to answer this question is to examine potential intermediate molecular phenotypes, and identify those modulated by genetic variants. In order to understand the pathogenic roles of CD4^+^ T_EM_ cells in autoimmunity, we aimed to characterize the variation in their phenotypic and functional markers in a healthy population, and to identify whether these markers intersect with the genetic basis for autoimmunity.

The majority of autoimmune disease risk variants are located in non-coding regions of the genome. It is reasonable to hypothesize that a subset of them causes disease by altering gene regulatory mechanisms as expression quantitative trait loci (eQTL) [6]–[9]. So far, studies of gene regulation have largely been carried out in cell lines and primary resting blood cells including undifferentiated CD4^+^ T cells, B cells, monocytes, and dendritic cells [8], [10]–[12]. However, to understand the pathogenic mechanisms of risk variants, especially when studying the immune system where cells are highly diverse and functionally specialized, it is crucial to focus on relevant cell types and stimulated cellular states.

We have previously shown that genes within RA risk loci were most specifically expressed in CD4^+^ T_EM_ cells, compared to more than 200 other immune cell types of various lineages and developmental stages (*p* = 1.00×10^−8^; **Figure S1**) [13]. Celiac disease and T1D loci were also enriched for genes specifically expressed in CD4^+^ T_EM_ cells (*p* = 1.43×10^−5^ and 1.29×10^−4^, respectively; **Figure S1**) [13]. Non-coding single nucleotide polymorphisms (SNPs) associated with RA significantly overlap chromatin marks of trimethylation of histone H3 at lysine 4 (H3K4me3) specifically in CD4^+^ regulatory and memory T cells (*p* = 1.3×10^−4^ and 7.0×10^−4^, respectively) [14].

We hypothesized that the risk alleles of these conditions might influence CD4^+^ T_EM_ quantitative molecular phenotypes: 1) the expression of immune-related genes; 2) the relative abundance of CD4^+^ T_EM_ cells in peripheral blood; and 3) proliferative response to T cell receptor (TCR) stimulation. To this end, we undertook a large immunoprofiling study in a healthy population of 174 European-descent individuals, by cross-analyzing genotype, transcription, abundance, and proliferative response in primary CD4^+^ T_EM_ cells. Because the post-stimulation activation of CD4^+^ T_EM_ cells is presumably crucial for their autoimmune response, we assayed cells not only at rest, but also after T cell receptor (TCR) stimulation with anti-CD3/CD28 beads. As such, this study is the first to our knowledge to map expression quantitative trait loci and examine immunological cellular traits in primary CD4^+^ T_EM_ cells under multiple states.

Using the ImmunoChip platform, investigators recently densely genotyped 186 loci disease that originally arose through genome-wide association studies (GWAS) in case-control samples for RA, CeD, and inflammatory bowel disease [15]–[17], as well as T1D (unpublished data). Dense genotyping allowed localization of association signals within these disease loci to a set of alleles that are very likely to be causal. Within these loci, we have a greater ability to identify co-localization between alleles driving variation in molecular phenotypes (such as eQTLs) and the disease risk alleles. However, in instances where multiple variants are in perfect linkage, we cannot pinpoint the exact causal variant without functional evaluation.

## Results

**Figure 1:**
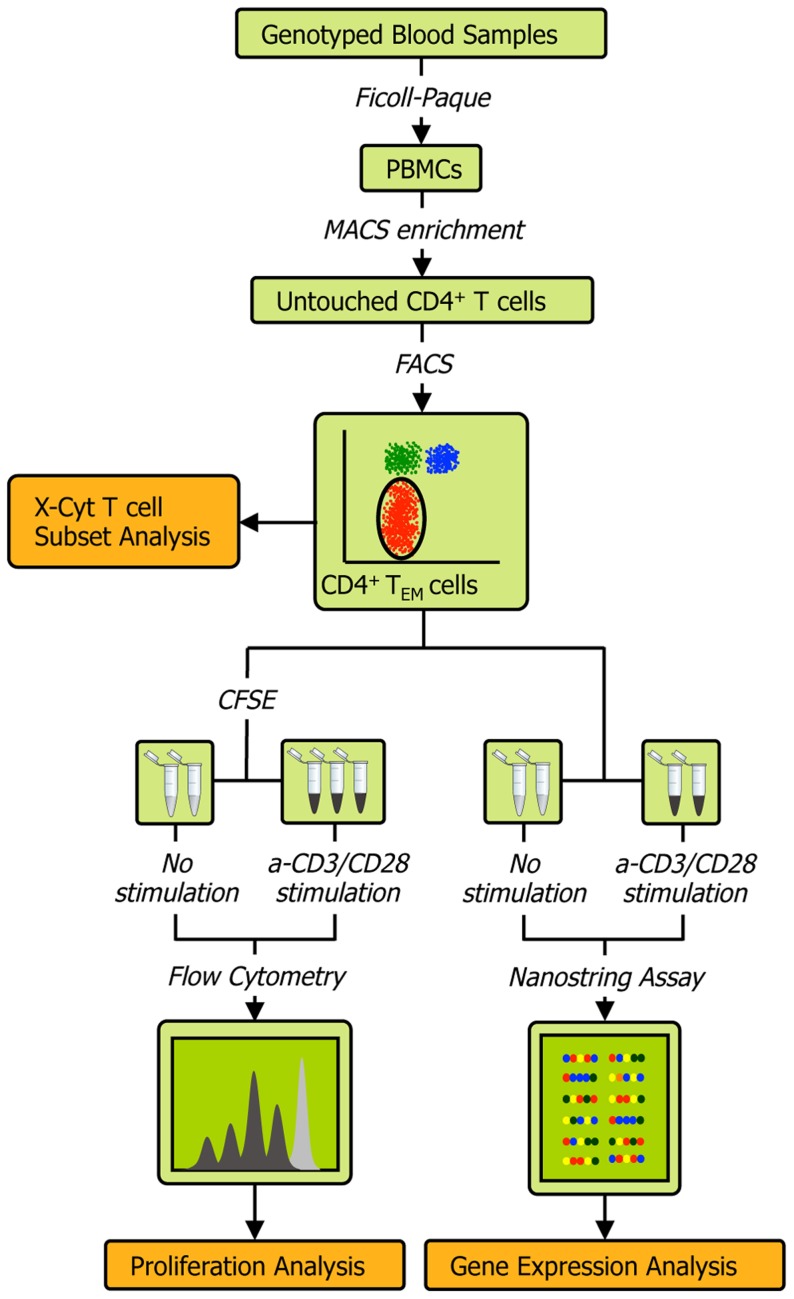
Schematic of the experimental workflow. We collected four types of data from each individual: 1) quality-controlled genome-wide SNP data containing 638,347 markers collected on Illumina Infinium Human OmniExpress Exome BeadChips, 2) abundance of CD4 T_EM_ cells as a percentage of all CD4 T cells obtained by FACS and quantified by X-Cyt, 3) average cell division upon T cell receptor stimulation by anti-CD3/CD28 commercial beads, measured using a CFSE (carboxyfluorescein succinimidyl ester) dye dilution assay, and 4) expression of 215 genes measured by NanoString nCounter. We repeated each proliferation assay in two-three technical replicates. Cell sorting purity and replication correlations for CD4 T_EM_ abundance, division index, and proliferation index are shown in **Figure S2**.

The experimental protocol (**Figure 1**) is described in detail in **Methods** and **Text S1**. Briefly, we obtained peripheral blood mononuclear cells (PBMCs) from the whole blood of healthy individuals via Ficoll-Paque centrifugation, and then used magnetic- and fluorescence-activated cell sorting to isolate CD4^+^ T_EM_ cells at a high degree of purity (>90%; see **Figure S2A**). We acquired genome-wide genotype data of about 640,000 SNPs on Illumina Infinium Human OmniExpress Exome BeadChips [18]. For each individual we then measured three quantitative phenotypes: 1) the expression of 215 genes (see **Table S1**) before and after T cell receptor (TCR) stimulation by anti-CD3/CD28 antibody beads; 2) the relative abundance of CD4^+^ T_EM_ cells (CD45RA^−^/CD45RO^+^/CD62L^−/low^) as a proportion of total CD4^+^ T cells; and 3) proliferation upon stimulation. Since we had low numbers of primary cells for expression profiling, we used the highly sensitive NanoString nCounter assay to avoid biases potentially induced by cDNA preparation. Out of the 215 genes assayed, 115 were within densely genotyped disease risk loci (see **Tables S2 and S3**). We quantified CD4^+^ T_EM_ cell abundance with X-Cyt, an automated statistical method that accurately identifies cell populations in cytometry data [19].

### Mapping cis-eQTLs that regulate genes in risk loci

We first aimed to identify SNP variants that regulated expression of genes in *cis*. To best localize eQTL signals, we imputed 1000 Genomes variants within 250 kb from the transcription start site (TSS) of each gene (excluding five HLA genes and five long non-coding RNAs). We tested SNPs in gene-coding and non-coding regions in both resting and stimulated CD4^+^ T_EM_ cells. We included gender and the top five principal components of the genotype data (calculated by EIGENSTRAT) as covariates in regression. To adjust for multiple hypothesis testing, we conducted 10,000 permutations within each gene region to calculate empirical *p*-values, and then reported associations at a false discovery rate of 5%.

**Figure 2:**
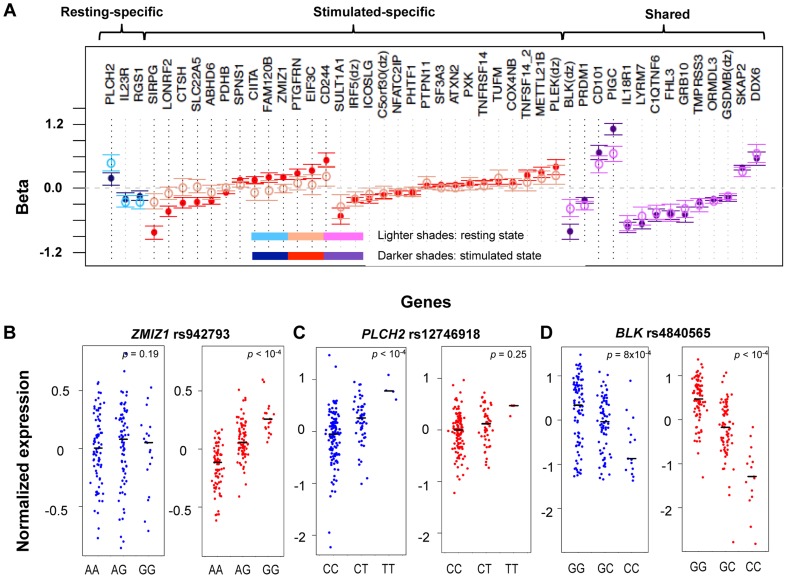
State-specific effects of eQTL SNPs. **A**) For a subset of genes, the correlation effects (β) of the top associated SNP across resting and stimulated cells differed. The genes shown with a black-dotted vertical lines had significantly different effect sizes across states. Black horizontal segments in **B**)**–D**) denote median values. Blue panels show resting-state (normalized) expression values; red panels show stimulated expression values. **B**) rs942793^G^ significantly increased the expression of *ZMIZ1* only in stimulated cells. **C**) rs12746918^T^ was correlated with increased expression of *PLCH2* in resting cells only. **D**) rs4840565^C^ decreased *BLK* expression in stimulated cells nearly twice as much as in resting cells [*β*_rest_(SE) = −0.366(0.085), *β*_stim_ = −0.805(0.071)].

In total, we observed 46 genes (22.4%) with *cis*-eQTL signals, including 17 in resting cells and 43 in stimulated cells (**Tables 1** **and** **2** **,** **Figure 2A**). For 14 of the 46 genes (30.4%), we detected eQTL signals in both resting (14/17, 82.4%) and stimulated (14/43, 32.6%) states. In four of these 14 genes (*FHL3, GRB10, IL18R1, and PIGC*), the lead eQTL SNPs across resting and stimulated states were identical. In another five genes (*C1QTNF6, PRDM1, SKAP2, DDX6, and LYRM7*), the lead SNPs are in tight LD (*r* ^2^ = 0.80∼1; based on 1000 Genomes Release 2, European samples). For the remaining five genes (*BLK, TMPRSS3, CD101, ORMDL3, and GSDMB*), the lead SNPs from the two states were in partial LD (0.42<*r* ^2^<0.56). In these five cases, we could not be confident that the eQTL SNPs across stimulation states were tagging the same variant.

**Table 1:** Cell-state specific eQTLs.

				eQTL Effect		Rest			Stim				
Chr	Top SNP	Minor allele	Gene	Rest	Stim	Fold Δ per Allele	Assoc. p-value	FDR	Fold Δ per Allele	Assoc. p-value	FDR	pΔ*	CD4 TEM-specific
1	rs12746918	T	*PLCH2*	X		1.61	4.75E-09	<0.001	0.18	2.35E-03	>0.20	2.70E-03	
1	rs10789226	G	*IL23R*	X		0.78	1.05E-05	0.022	−0.21	2.34E-03	>0.20	6.78E-01	Yes
1	rs6704162	G	*RGS1*	X		0.77	2.96E-05	0.036	−0.14	1.71E-03	>0.20	1.25E-01	
20	rs3746721	A	*SIRPG*		X	−0.24	1.67E-03	>0.20	0.44	3.29E-28	<0.001	1.91E-09	
2	rs11123823	A	*LONRF2*		X	−0.09	1.52E-01	>0.20	0.65	7.09E-17	<0.001	4.26E-06	
5	rs10058074	A	*SLC22A5*		X	0.04	5.35E-01	>0.20	0.78	1.81E-09	<0.001	1.11E-04	
10	rs942793	G	*ZMIZ1*		X	0.00	9.85E-01	>0.20	1.23	3.67E-21	<0.001	7.70E-07	Yes
16	rs143150526	A	*SULT1A1*		X	−0.35	2.60E-03	>0.20	0.59	4.04E-10	<0.001	2.08E-01	Yes
3	rs1399754	A	*ABHD6*		X	−0.08	2.61E-01	>0.20	0.79	1.11E-12	<0.001	4.07E-02	
7	rs3807306	G	*IRF5*		X	−0.19	3.32E-03	>0.20	0.80	3.07E-15	<0.001	7.41E-01	
5	rs39984	T	*C5orf30*		X	−0.09	1.77E-01	>0.20	0.87	1.90E-08	<0.001	4.36E-01	Yes
1	rs12138115	G	*SF3A3*		X	0.02	3.29E-01	>0.20	1.06	1.63E-08	<0.001	7.10E-02	
1	rs6671426	A	*TNFRSF14*		X	0.06	1.18E-01	>0.20	1.12	1.28E-09	<0.001	1.89E-01	
16	rs7140	C	*SPNS1*		X	0.07	8.07E-03	>0.20	1.16	2.73E-12	<0.001	3.10E-02	
12	rs1021469	A	*METTL21B*		X	0.20	2.72E-03	>0.20	1.35	1.22E-09	<0.001	2.09E-01	Yes
2	rs10167650	G	*PLEK*		X	0.25	6.30E-03	>0.20	1.49	4.98E-08	<0.001	2.11E-01	
1	rs11265501	A	*CD244*		X	0.23	1.50E-02	>0.20	1.70	1.46E-12	<0.001	1.06E-02	
15	rs7183668	G	*CTSH*		X	0.02	7.63E-01	>0.20	0.76	2.19E-07	0.001	1.55E-03	
16	rs6498114	A	*CIITA*		X	−0.07	3.70E-01	>0.20	1.17	6.38E-07	0.001	7.36E-03	
3	rs149241987	T	*PDHB*		X	0.01	6.61E-01	>0.20	0.93	1.94E-07	0.001	1.28E-02	
16	rs11639897	C	*TUFM*		X	0.20	1.21E-02	>0.20	1.13	3.05E-07	0.001	3.43E-01	
1	rs4659344	G	*PTGFRN*		X	0.10	1.27E-01	>0.20	1.31	1.18E-07	0.001	3.09E-02	
16	rs146435192	T	*EIF3C*		X	0.07	4.84E-01	>0.20	1.38	4.06E-06	0.002	2.96E-02	Yes
1	rs971173	T	*PHTF1*		X	−0.06	1.24E-01	>0.20	0.92	7.76E-06	0.004	7.24E-01	
3	rs11711261	A	*PXK*		X	0.10	1.12E-01	>0.20	1.11	5.72E-06	0.004	9.36E-01	
21	rs2847224	A	*ICOSLG*		X	−0.11	5.22E-02	>0.20	0.82	1.44E-06	0.005	1.90E-01	
12	rs11066028	C	*ATXN2*		X	0.01	6.64E-01	>0.20	1.07	1.86E-05	0.005	1.63E-01	
12	rs3858706	C	*PTPN11*		X	0.11	2.81E-01	>0.20	1.05	2.17E-05	0.009	3.65E-01	Yes
19	rs2291668	A	*TNFSF14*		X	0.12	9.21E-02	>0.20	1.28	5.16E-06	0.017	2.44E-01	
16	rs8587	A	*COX4NB*		X	0.07	1.96E-01	>0.20	1.13	8.44E-06	0.018	4.28E-01	
16	rs7498329	C	*NFATC2IP*		X	−0.07	3.85E-03	>0.20	0.91	9.22E-05	0.037	4.53E-01	Yes
6	rs7453655	A	*FAM120B*		X	−0.04	7.02E-01	>0.20	1.22	3.83E-05	0.042	2.41E-02	

For each row we list a SNP and the gene transcript for which it is a *cis*-eQTL. We indicate whether the effect is observed in resting or stimulated CD4+ effector memory T cells. For each SNP we indicate the fold change in expression conferred per allele, assuming an additive model and the false discovery rate estimate. Finally, we indicate whether the effect is specific for CD4+ effector memory T cells; we define specificity as the absence of any cis-eQTL effect in PBMCs at FDR>50% [8].

* pΔ measures the difference between the effect sizes across resting and stimulated states.

**Table 2:** Genes with resting and stimulated eQTLs.

		Resting state					Stimulated state							
Gene	Chr	SNP_rest	minor allele	Fold Δ per Allele	assoc. P-value	FDR_rest	SNP_stim	minor allele	Fold Δ per Allele	assoc. P-value	FDR_stim	R2	pΔ**	CD4 TEM-specific
**BLK**	8	rs4840565	C	0.69	2.75E-05	0.012	rs4840565	C	0.45	1.35E-22	<0.001	1*	7.38E-05	
**C1QTNF6**	22	rs229515	T	0.63	6.10E-10	<0.001	rs229522	A	0.61	8.75E-15	<0.001	1	5.41E-01	
**CD101**	1	rs4620527	G	1.80	3.06E-09	<0.001	rs9332416	A	1.96	1.76E-19	<0.001	0.547	3.23E-02	yes
**DDX6**	11	rs500254	G	1.92	2.33E-13	<0.001	rs4938544	A	1.75	1.48E-16	<0.001	0.834	3.40E-01	yes
**FHL3**	1	rs67631072	T	0.63	6.43E-08	<0.001	rs67631072	T	0.61	9.53E-26	<0.001	1*	7.05E-01	
**GRB10**	7	rs12536500	T	0.68	4.10E-06	0.016	rs12536500	T	0.61	4.92E-11	<0.001	1*	3.11E-01	
**GSDMB**	17	rs36038753/rs12936409	T	0.86	2.20E-07	<0.001	rs36038753/rs12936409	T	0.83	2.64E-12	<0.001	1*	3.65E-01	
**IL18R1**	2	rs3771164	T	0.52	6.35E-16	<0.001	rs3771164	T	0.48	3.79E-31	<0.001	1*	4.35E-01	
**LYRM7**	5	rs12522164	A	0.59	1.05E-08	<0.001	rs12517633	T	0.51	1.38E-29	<0.001	0.792	1.36E-01	
**ORMDL3**	17	rs35222145	G	0.75	1.16E-19	<0.001	rs2290400	C	0.82	4.44E-28	<0.001	0.482	2.75E-01	
**PIGC**	1	rs1063412	A	1.91	1.36E-14	<0.001	rs1063412	A	3.04	2.65E-46	<0.001	1*	6.79E-07	
**PRDM1**	6	rs811925	G	0.72	2.61E-13	<0.001	rs578653	G	0.81	3.30E-13	<0.001	0.944	2.78E-02	
**SKAP2**	7	rs4719882	G	1.37	1.85E-12	<0.001	rs3801813	C	1.46	6.08E-39	<0.001	0.862	1.17E-01	
**TMPRSS3**	21	rs7283281	G	0.76	1.08E-06	0.005	rs2277798	A	0.77	7.74E-10	<0.001	0.55	5.42E-01	

As in Table 1, for each row we list a SNP and the gene transcript for which it is a *cis*-eQTL. For each SNP we indicate the fold change in expression conferred per allele, assuming an additive model and the false discovery rate estimate. Finally, we indicate whether the effect is specific for CD4+ effector memory T cells [8].

* denotes identical SNPs.

** In cases where the lead resting and stimulated SNPs differ, pΔ compares the cross-state effect sizes of only one SNP. For each gene, the SNP with the stronger association across the two states was considered.

Three genes (*IL23R, PLCH2, and RGS1*) had statistically significant eQTLs exclusively in the resting state, while 29 genes had statistically significant eQTLs exclusively in stimulated cells, such as rs942793 associated with *ZMIZ1* expression (**Figure 2B**). One possibility is that some SNPs failed to reach significance threshold due to the small sample size or low expression levels in resting cells. However, we observed many genes with truly state-specific eQTLs, where the estimated effect sizes (*β*) of the eQTL SNP differed significantly across resting and stimulated states. To systematically compare the *β*_rest_ and *β*_stim_ for each gene, we used a *z*-statistic to quantify the probability that they differ. We then reported the *p*-value (two-tailed) assuming that *z* is distributed as standard normal, considering p<0.05 to be significantly different (“state-specific”; see **Tables 1** **and** **2**). For example, rs12746918^T^ increased the expression of *PLCH2* significantly only in resting cells; and *β*_rest_ was approximately twice as large as *β*_stim_ (**Figure 2C**). We note that 1 of the 3 eQTLs in resting cells was state-specific (p<0.05), and 13 out the 29 eQTLs seen in stimulated cells were state-specific (p<0.05). Of the 14 eQTLs that were shared between resting and stimulated cells, only 4 of them, *BLK* (**Figure 2D**), *CD101*, *PIGC*, and *PRDM1*, had different *β's* across states. The abundance of eQTLs detected exclusively in stimulated cells underscores the importance of studying cells in different cellular states.

We wanted to assess whether the eQTLs might act by altering gene regulatory elements in CD4^+^ T_EM_ cells. To this end we asked whether the eQTL SNPs co-localized with marks of active promoters or enhancers. We utilized H3K4me3 marks from the NIH Roadmap Epigenomics Mapping Consortium [20] measured by ChIP-seq in primary CD4^+^ memory T cells. For the SNP with the strongest association to each gene, we queried the distance of the nearest H3K4me3 mark to this SNP or its LD partners (*r* ^2^>0.8). We compared this distance measure between two sets of SNPs: the 46 SNPs with significant eQTL associations (FDR<5%, resting or stimulated), and the SNPs most strongly correlated with the other 159 genes but did not reach significance threshold. Indeed, the 46 significant eQTL SNPs were located at smaller distances to H3K4me3 marks (*p* = 1.10×10^−7^, one-sided Mann-Whitney test, **Figure S3A**). In addition, we queried the height of each H3K4me3 mark's peak, which reflected the number of reads at a given position compared to genomic controls as defined by the MACS software package. A tall peak gives us confidence that the mark is present in a large proportion of cells. Comparing the marks nearest to the two sets of SNPs, we saw that the 46 eQTL SNPs were also located near taller peaks (*p* = 9.56×10^−8^, **Figure S3B**).

### Many eQTLs are CD4^+^ T_EM_ cell-specific

We compared the *cis*-eQTLs we discovered to those found in heterogeneous peripheral blood mononuclear cells (PBMC) in a large genome-wide eQTL meta-study (n = 5,331) conducted by Westra *et al.* [8]. At 5% FDR, eleven of the 46 eQTL genes we identified showed no detectable signal in PBMCs at 50% FDR. We saw significant associations in 131 genes at 50% FDR, 53 of which had no signal in PBMCs at 50% FDR (**Tables 1** **and** **2**). We hypothesized that these genes tended to be more specifically expressed in CD4^+^ T_EM_ cells, thus making eQTLs readily detectable in the purified cell population. To assess this, we examined cell-specific expression of the genes the ImmGen dataset, which assayed the genome-wide expression in 247 murine mouse immunological cell types [13], [21]. We found that the genes with CD4^+^ T_EM_ cell-specific eQTLs (at 50% FDR) were more specifically expressed in CD4^+^ T_EM_ cells than genes with eQTLs detected in both datasets (*p* = 0.044, one-sided Mann-Whitney test).

### Autoimmune disease alleles affect the transcription of genes in cis

We then focused on 115 genes near 96 risk alleles of RA, T1D, and/or CeD in densely genotyped loci (182 gene-SNP pairs, including two risk alleles shared by at least two diseases, see **Tables S2 and S3**). We discovered that eleven (11.4%) disease-associated SNPs (6 of 24 RA SNPs, 5 of 37 T1D SNPs, and 3 of 37 CeD SNPs) correlated significantly with the expression of ten genes in either resting or stimulated state (**Table S3**). In addition, there was substantial enrichment of nominally significant associations (*p*<0.05) among disease SNPs. By random chance, we expected about nine SNP-gene pairs to reach nominal association in each stimulation state. However, we observed 26 pairs (14.2%) with nominal association in resting cells (*p* = 4.67×10^−7^, one-tailed binomial test). Even more strikingly, we observed 45 pairs (24.7%) with nominal association in stimulated cells (*p*<10^−15^, one-tailed binomial test).

**Figure 3:**
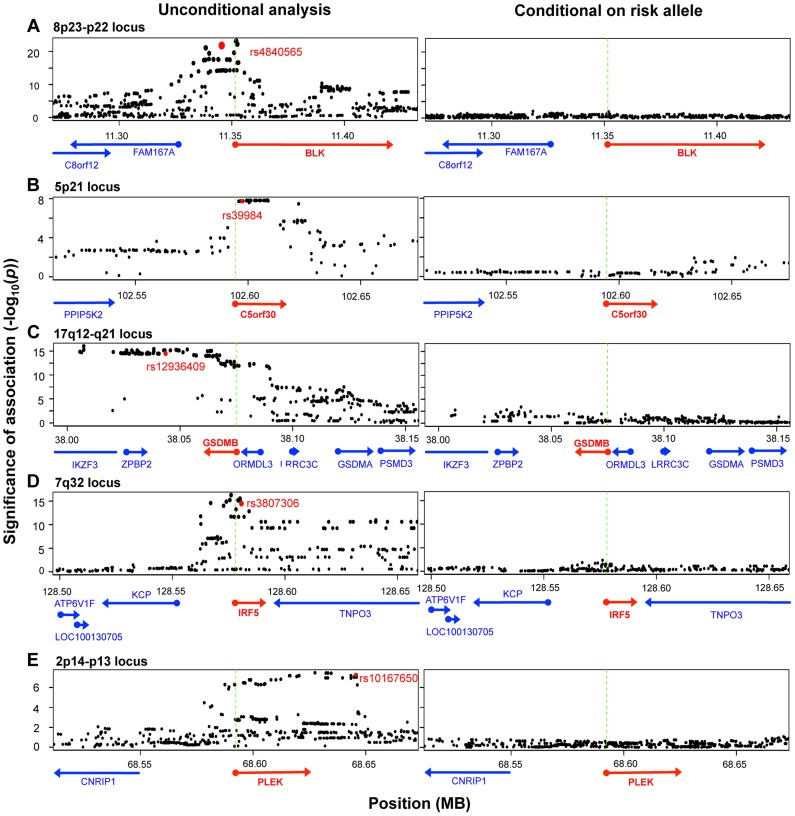
Five disease risk alleles explained the eQTL associations with five genes. The left-sided panels show unconditional SNP-expression association results. Green dashed lines mark the TSS of the eQTL gene. The red dots indicate the risk alleles associated with the expression of respective genes shown as red arrows. The right-sided panels show adjusted association results after conditioning for the respective risk alleles. In each of the five loci, conditioning on the disease SNP obviated signals in the entire region, such that no association more significant than *p* = 0.05 remains.

To identify those instances where the disease-associated SNP could explain the entire eQTL signal in the gene region, we applied conditional analysis to identify any residual signals after controlling for the disease SNP. In five of the ten genes (*BLK, C5orf30, GSDMB, IRF5, PLEK*), conditioning on the disease SNP obviated any remaining eQTL signal in the region (no SNP with permutation *p*-value <0.05; **Figure 3**), suggesting that there was a single variant (the disease-associated SNP or one in very high LD to it) that drove variation in expression. Interestingly, as previously noted, the lead SNPs in resting and stimulated states for *BLK* and *GSDMB* were in partial linkage to each other. The absence of residual eQTL signal upon conditioning on the same risk allele might suggest that the lead SNPs were indeed tagging the same causal SNP in each of these genes. In each of the other five genes (*ORMDL3*, *SKAP2*, *TMPRSS3*, *TNFRSF14*, and *ZMIZ1*), evidence of independent eQTL effect remained after conditional analysis. In these instances the disease-associated SNP and remaining lead signal are in partial linkage disequilibrium (*r* ^2^ = 0.36–0.73). In these cases, we could not conclude whether the disease SNPs drove the alteration in expression, or whether the true causal SNPs were in partial linkage and caused spurious associations. It is probable that disease risk alleles were indeed causal, yet we could not confidently fine-map the effect due to experimental noise in expression assays or inadequate sampling.

We note that another 26 genes within disease loci associated contained *cis*-eQTL signals, but that these *cis*-eQTL signals did not co-localize with RA, T1D, or CeD alleles. As these loci had been fine-mapped using Immunochip, the lack of overlap strongly suggested that these *cis*-eQTLs and disease-causing variants were distinct. For example, rs798000 is an RA risk allele located in a non-coding region upstream of *CD2*, *CD58*, and *PTGFRN*. However, it was not associated with the expression of any of these genes (*p*>0.5). Another example was rs6911690, an RA allele located about 60 kb 5′ of *PRDM1*, that was not associated with the expression of the gene at rest or after stimulation (*p*>0.5). The lead eQTL SNP associated to *PRDM1* was rs578653 (FDR<10^−3^), which was not in LD with the disease allele (r^2^<0.05).

### The genetic basis of CD4^+^ T_EM_ cell proliferation

The relative peripheral abundance of CD4^+^ T_EM_ cells varied between individuals (mean = 9.57%; SD = 4.85%), and was reproducible 35 individuals with two separate blood draws more than one month apart (Pearson's *r* = 0.87, *p* = 1.77×10^−11^, see also **Figure S2B**). Consistent with other studies, we observed that the relative proportion of CD4^+^ T_EM_ cells increased with age by 0.11% per year (*p*_age_ = 1.92×10^−3^) [22]. We also observed that on average men had 2.22% more CD4^+^ T_EM_ cells than women (*p*_gender_ = 3.80×10^−2^; see **Figure S4**). Upon anti-CD3/CD28 stimulation, there was a substantial inter-individual variation in proliferation measured by both division index (DI, average number of divisions undergone by all cells; mean = 1.46, SD = 0.35), and proliferation index (PI, average number of divisions undergone only by dividing cells; mean = 2.16, SD = 0.21). Proliferation metrics were also reproducible in the 35 individuals (Pearson's *r_DI_* = 0.57; Pearson's *r*_PI_ = 0.62, **Figures S2C and S2D**). Interestingly, proliferation was negatively correlated to the proportion of CD4^+^ T_EM_ cells (*p_DI_* = 1.28×10^−3^, *p_PI_* = 1.93×10^−3^), but was not associated to age or gender (*p*>0.3). This negative correlation needs to be replicated in an independent dataset. Effector functions of T_EM_ cells with higher proliferative capacities need to be examined to understand whether they represent a hyperactive subset whose abundance is controlled to maintain immune homeostasis. Possibly individuals with a lower proportion of T_EM_ cells are relatively enriched for these subsets.

**Figure 4:**
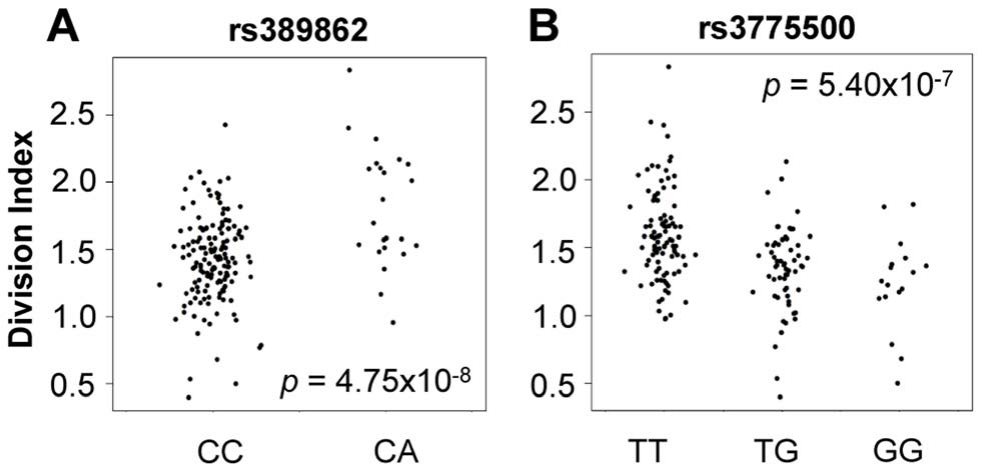
Genome-wide association to division index (the average number of division undergone by all cells). **A**) rs389862^A^ on chromosome 13 was significantly associated to increased division index at *p* = 4.75×10^−8^, and is located in a non-coding region 30 kb upstream of *RASA3*, and 70 kb upstream from *CDC16*. **B**) rs3775500^G^ on chromosome 4 shows a strongly suggestive association at *p* = 5.40×10^−7^, and is located within the *DAPP1* (Bam32) gene.

We tested genome-wide SNPs for association to relative abundance, division index, and proliferation index, considering *p*<5×10^−8^ as the threshold for significance. For abundance, we included gender, age, and the top five principal components of genotypes as covariates. Given the correlation with proliferation, we also included the measured CD4^+^ T_EM_ relative abundance as an additional covariate. We observed associations to division index in several loci, including 13q34 led by rs389862 (*p* = 4.75×10^−8^; **Figure 4A**). This SNP is a non-coding variant located 30 kb upstream of *RASA3*, and 70 kb upstream from *CDC16*. Both genes have known roles in regulating cell proliferation or differentiation [23], [24]. This SNP was also strongly associated with proliferation index (*p* = 2.75×10^−7^). Additionally, there was a strongly suggestive association to rs3775500 on chromosome 4, located in the intron of *DAPP1*, which encodes the Bam32 protein (*p* = 5.40×10^−7^; **Figure 4B**), which is an adaptor protein expressed solely in antigen presenting B cells. Interestingly, mutations in this gene have been shown by several groups to affect T cell activation [25], [26], suggesting the possibility that B cells may indirectly regulate T cell function in autoimmunity. We did not observe any significant association with the relative abundance of CD4^+^ T_EM_ cells.

**Figure 5:**
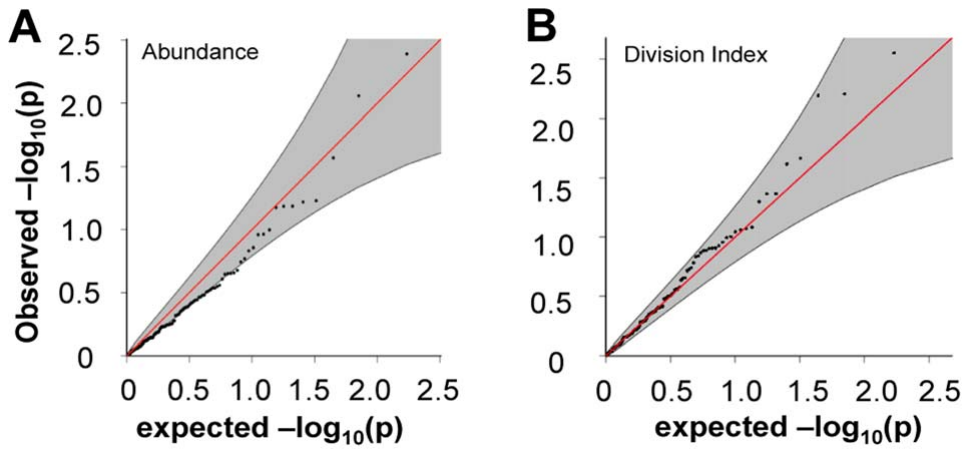
Risk alleles of CeD, RA, and T1D, showed no significant association to CD4 T_EM_ cell abundance or proliferation. **A**) The 118 SNPs with association to diseases in densely genotyped regions on Immunochip platform were not significantly associated to CD4 T_EM_ cell abundance. The shaded region shows 95% confidence interval. See also **Figure S5**. **B**) The same set of 118 risk alleles also showed no inflation in association with proliferative response measured as division index.

When we extracted the association statistics of 118 densely genotyped risk alleles of CeD, RA, and/or T1D, they showed no inflation in association *p*-values for relative abundance of CD4^+^ T_EM_ cells (**Figure 5A** **, Table S2**). This suggested that risk variants did not modify risk via modulation of CD4^+^ T_EM_ peripheral abundance. We recognized that the power to detect significant associations might have been limited in our study by the sample size. However, this negative finding was corroborated by results from a recently published study with data from ∼2800 individuals, in which the same set of risk alleles also showed no significant association to CD4^+^ T_EM_ (see **Figure S5**) [27]. Similarly, the same set of risk alleles did not show significant association to proliferative response (**Figure 5B** **, Table S2**). Based on these data, it was unlikely that SNP variants associated to RA, T1D, or CeD conferred risk through modulation of CD4^+^ T_EM_ cell abundance or proliferation.

### Gene expression in resting cells predicted post-TCR stimulation proliferation

After stimulation we observed that 122 genes showed significant changes in expression in response to stimulation, including 78 whose expression at least doubled or decreased by 50% (**Table S1**). The gene with the greatest post-stimulation induction was *GZMB* (average fold change = 93.48), which encodes granzyme B, a protein involved in the apoptosis of target cells during cell-mediated immune response in cytotoxic and memory lymphocytes. The most significantly down-regulated gene was *GRB10* (average fold change = 0.18), which is near rs6944602 associated with T1D and encodes growth factor receptor-bound protein 10, whose function in the immune system is unclear.

**Figure 6:**
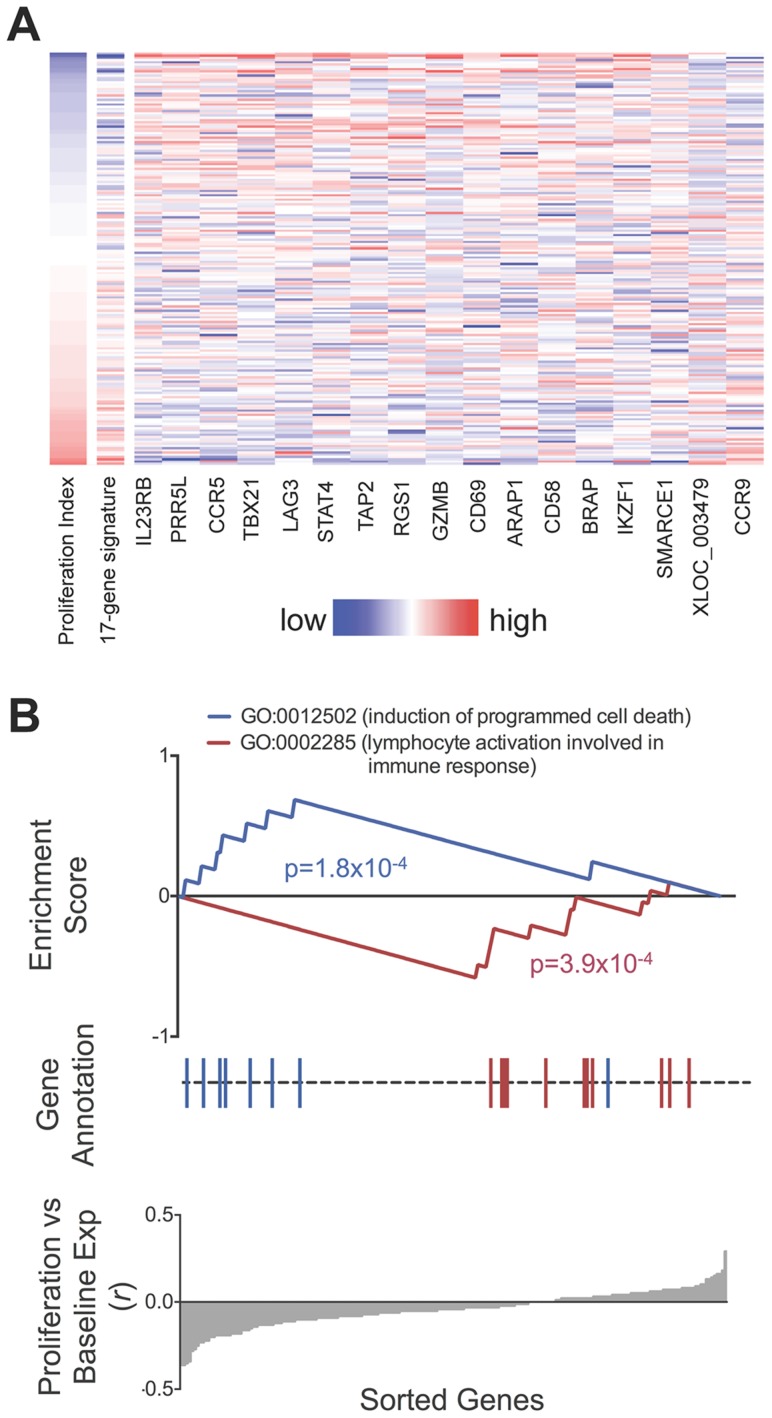
Relationship between baseline expression and post-stimulatory response. **A**) Baseline expression of 17 genes correlated with post-stimulation proliferation. Rows in the heatmap are ordered from top to bottom by ascending proliferation index. Genes/columns are ordered from the most negatively correlated (IL23RB) to the most positively correlated (CCR9). The 17-gene signature was calculated as the weighted sum of the 17 genes, where the negatively-correlated genes were given a weight of −1, and the positively-correlated genes were given a weight of +1. **Table S1** lists the correlation coefficient and p-value for each gene. **B**) Genes correlated with proliferative response were enriched for apoptosis and lymphocyte activation pathways. Genes correlated to lower proliferative response (proliferative index) were enriched for Gene Ontology code GO:0012502 (induction of programmed cell death, p = 1.8×10^−4^). Conversely, genes correlated to higher proliferative response were enriched for GO:0002285 (lymphocyte activation, p = 3.9×10^−4^).

We observed that relative gene expression at rest predicted proliferative response. In 182 individuals with both proliferation and gene expression data, 17 of the 215 genes were associated with proliferation index (*p*<0.01, two-tailed test by permuting proliferation data, **Figure 6**, **Table S1**). Increased expression of 15 of the 17 genes including *CCR5, IL2RB, PRR5L, and TBX21*, were correlated with reduced proliferative response, while *CCR9* and the lncRNA XLOC_003479 showed significant correlation with increased proliferation. This number of correlated genes was far in excess of random chance based on a null distribution consisting of 1000 permutations (*p*<10^−3^, median 2, maximum 15). The weighted sum of the 17 genes served as a “proliferation potential signature”, where we weighted the positively- and negatively-correlated genes as +1 and −1, respectively. This signature strongly predicted proliferation index (r = 0.55). We show the correlation between each of the 17 genes as well as the aggregate signature to proliferation as a heatmap (**Figure 6A**). To assess if we were overfitting the data, we applied a two way cross-validation, where we defined the proliferation signature based on genes from half of the individuals and tested correlation to proliferation in the remaining half of the individuals. In both instances we again observed significant prediction of proliferation (r = 0.41,one tailed p<10^−3^ by permutation; r = 0.39, p<10^−3^).

To search for biological pathways underlying genes correlated to proliferation, we applied gene set enrichment analysis (GSEA) to test for enrichment for 1,008 functional gene sets based on Gene Ontology codes [28] (**Figure 6B**). Genes correlated to reduced proliferation were most significantly enriched for GO:0012502 (induction of programmed cell death; one tailed *p* = 1.8×10^−4^); those correlated with increased proliferation were most significantly enriched for GO:0002285 (lymphocyte activation involved in immune response, one tailed *p* = 3.9×10^−4^).

Using data from 29 individuals each with two samples collected at least one month apart, we replicated the observed correlation. In these samples we performed a cross-visit analysis, and observed that the same 17-gene signature from the first visit significantly predicted proliferation indices on the second visit (*r* = 0.65, *p* = 0.0006, 1-tailed permutation), and *vice versa* (*r* = 0.55, p = 0.0019).

## Discussion

To fine-map and link risk loci to their pathogenic mechanisms, we investigated molecular and immune phenotypes potentially leading to disease end-points. The immune system is particularly complex, and different cells under various activation states have specialized functions that may not be adequately captured by examining PBMCs. Therefore, we focused on one purified cell population that had been shown to be important for the pathogenesis of several autoimmune diseases. We quantified population variation in several traits, including peripheral abundance, proliferative response to TCR stimulation, and expression of genes within autoimmune disease loci at rest and after stimulation. In **Tables 1**, **2**, and **S2**, we provide significant cis-eQTLs and genome-wide association results.

To our knowledge this study was a first cross-examination of genetic-, transcriptional-, and cellular-level quantitative traits in CD4^+^ T_EM_ cells. It demonstrated the importance of focusing functional studies in a purified cell population under relevant developmental and stimulation states. By examining the proliferative response upon TCR stimulation, we identified a subset of genes whose baseline expression predicted proliferative potential. Intriguingly, these genes were involved in programmed cell death and lymphocyte activation. Whether variation in proliferative abilities correlated with cytokine production and other signaling functions, thus affecting susceptibility to autoimmunity, remains a question to be addressed by future studies.

Of the 205 genes in disease loci that we examined, 46 had *cis*-eQTLs. Notably, eleven of these were specific to stimulated CD4^+^ T_EM_ cells, and not previously found in PBMCs. We noted that approximately 10% of genes within risk loci of diseases had *cis*-eQTLs. However in many instances the lead eQTL SNPs were unrelated to the disease-associated SNPs. One example of a disease allele that functioned as *cis*-eQTL was rs39984, which was associated to lower risk of RA, and regulated the expression of *C5orf30* encoding an UNC119-binding protein. This SNP variant is located in the first intron of *C5orf30*, and indeed explained the entire *cis*-eQTL signal in this gene (see **Figure 2B**). This eQTL effect was previously undetected in PBMCs, and the protein's functional role in the immune system is largely unknown. However, a recent study showed that rs26232 (the lead GWAS SNP prior to fine-mapping, *r^2^* = 0.988 to rs39984) was correlated with lower severity of radiologic damage in RA, independent of previously established biomarkers [29]. Another gene in the locus, *GIN1*, is located 140 kb from rs39984; however its expression showed no correlation with the SNP (*p*>0.5).

Another CD4^+^ T_EM_ cell-specific eQTL gene was *DDX6*, which encodes DEAD-box RNA helicase 6. However, in this case, the lead eQTL SNP (rs4938544) associated to increased expression of *DDX6* in stimulated cells was not in LD with the known CeD risk allele (rs10892258, *r* ^2^<0.1) or the RA risk allele (rs4938573, *r* ^2^<0.1). Neither risk allele showed significant association to DDX6 expression (*p* = 0.19 and 0.26, respectively). Both risk alleles are also located near *CXCR5*, *BCL9L*, and *TREH*; none of these genes had reported *cis*-eQTLs in PBMCs [8]. However, we did not assay these three genes in this study, therefore could not confirm the role of disease alleles in regulating their expression in CD4^+^ T_EM_ cells.

Although we did not assay all genes or test for *trans*-acting eQTLs, based on the level of co-localization between eQTL SNPs and risk alleles observed in the study, we found it unlikely that all non-coding risk variants caused disease by altering gene expression within resting or stimulated CD4^+^ T_EM_ cells. In addition, while changes in proportions of lymphocyte subsets had been observed in patients of autoimmune disorders [30]–[35], we did not find evidence to support disease alleles' roles in directly modulating CD4^+^ T_EM_ cell abundance or proliferative response. Ultimately, other cell states and cell types will need to be investigated.

We recognize several limitations to the current study. In order to conduct a focused study on a small amount of purified primary cells we used the NanoString nCounter assay system. This avoided potential biases and artifacts arising from cDNA synthesis required for microarray or RNA-seq studies, but restricted our analysis to a subset of candidate genes within risk loci of CeD, RA, and T1D, rather than a genome-wide expression analysis. Consequently we could not identify trans-eQTLs, splice variants, or epistatic effects on expression regulation. Additionally, anti-CD3/CD28 stimulation for memory T cells is not antigenic, especially while in isolation from a “natural” multi-cellular environment, thus it was only partially physiological.

This and other cell-specific studies on population variation in molecular phenotypes are only a beginning of examining potential intermediate phenotypes. Post-activation cytokine production by CD4^+^ T_EM_ cells are likely crucial in driving autoimmunity. Therefore, it is critical that future studies of molecular phenotypes include proteomic assays to quantify functional markers of immune response. Finally, functional experiments will need to be conducted in the future to determine whether these molecular phenotypes are indeed intermediary to disease.

## Materials and Methods

### Ethics statement

All research was approved by our Institutional Review Board, and informed consent was obtained from each volunteer.

### Study sample

We enrolled 225 healthy volunteers (134 females, 91 males) of non-Hispanic Caucasian descent that proved informed consent through the Phenogenetics Project at Brigham and Women's Hospital. Subjects' ages ranged from 19 to 57 years with average female and male ages of 28.8 years and 34.9 years, respectively. Thirty-five subjects (18 females, 17 males) returned for a second study visit one to nine months after their initial visits.

### Genotyping

We genotyped each subject using the Illumina Infinium Human OmniExpress Exome BeadChip. In total, we genotyped 951,117 SNPs, of which 704,808 SNPs are common variants (minor allele frequency [MAF]>0.01) and 246,229 are part of the exome. After quality control, 638,347 common SNPs remained. Of all subjects, 174 subjects had abundance, proliferation, gene expression, and quality controlled genotype data. Detailed quality control criteria are described in **Text S1**.

### SNP imputation

For each gene, we selected a 500 kb region (250 kb each in the 3′ and 5′ directions) around the transcription start site and imputed 1000 Genomes SNPs into the genome-wide SNP data using BEAGLE Version 3.3.2. We used the European samples from 1,000 Genomes as the reference panel. We excluded markers that had MAF<0.05 in the reference panel as well as all insertion/deletions. After imputation, we excluded markers with a BEAGLE R^2^<0.4 or MAF<0.01 in the imputed samples.

### CD4^+^ T_EM_ cell isolation and stimulation

We isolated peripheral blood mononuclear cells (PBMC) from whole blood using a Ficoll density gradient (GE Healthcare). We then isolated CD4^+^ effector memory T cells from PBMCs first by magnetic-activated cell sorting to enrich for CD4^+^ T cells, followed by fluorescent-activated cell sorting using labeled antibodies against CD45RA, CD45RO, and CD62L.

We stimulated CD4^+^ T_EM_ cells by incubation with commercial anti-CD3/CD28 beads for 72 hours. For proliferation studies, we labeled cells with carboxyfluorescein diacetate succinimidyl ester (CFSE; eBioscience), and measured proliferation by dye dilution. Detailed isolation and purification methods are described in **Text S1** (also see **Figure S2A**).

### Gene expression

We designed the NanoString codeset based on GWAS SNPs associated with CeD, RA, and T1D as of April 2012. This list of SNPs can be found in **Supplementary Table S4**. As the numbers of associated loci with autoimmune diseases continuously expand, we refer the reader to ImmunoBase (https://www.immunobase.org) for up-to-date disease regions. For each locus, we defined a region of interest implicated by the GWAS lead SNP [36]. We identified the furthest SNPs in LD in the 3′ and 5′ directions (r^2^>0.5). We then extended outward in each direction to the nearest recombination hotspot. If no genes were found in this region, we extended an additional 250 kb in each direction. All genes overlapping this region were considered implicated by the locus. The final NanoString codeset (prior to expression data quality control) included 312 genes, including 270 genes near SNPs associated with 157 RA, CeD, and T1D through GWAS, 26 genes of immunological interest, and 15 reference genes with minimal change in expression after TCR stimulation (see **Supplementary Table S1**).

After quality control, 215 genes remained. Of all 225 subjects in the study, 187 subjects passed gene expression quality control for both resting and stimulated cells. Specific normalization and quality control procedures are described in **Text S1**.

### Genotype principal component analysis

To control for any potential population stratification, we adjusted all association tests using the top five principal components of our genome-wide SNP data. Principal components were generated via EIGENSTRAT using unsupervised analysis (no reference populations were used). The top five PCs explained 6.88% (2.08%, 1.27%, 1.20%, 1.17%, and 1.16%, respectively) of the total variance. After controlling for these five PCs, the lambda GC for CD4 T_EM_ proportion association was 1.008; that of division index was 1.001.

### Cis-eQTL analysis

For each gene-SNP pair, we applied linear regression using the first five principal components of the genotype data and gender as covariates. As such, normalized expression = β_0_+β_1_*allelic dosage+β_2*_PC_1_+β_3*_PC_2_+β_4*_PC_3_+β_5*_PC_4_+β_6*_PC_5_+β_7_*(factor)gender. To adjust for multiple hypothesis testing while taking into consideration the correlation among SNPs within each locus, we calculated a permutation-based *p*-value for each SNP. We performed 10,000 permutations of the residual expression values. We reported each SNP's *p*-value the proportion of permutation *P* value smaller than the analytical *p*-value. For conditional analysis, the vector of allelic dosages of the disease-associated SNP was included as an additional covariate.

### Quantification of CD4^+^ T_EM_ cells and proliferative response

We defined CD4^+^ T_EM_ cells as CD45RA^−^, CD45RO^+^, and CD62L^low/−^. In all samples CD4^+^ T_EM_ cells were quantified using *X-Cyt*, a mixture-modeling based clustering program for automated cell population identification (see **Figure S6**) [19]. We fit proliferation division peaks with one-dimensional Gaussian mixture models (see **Figure S7**). Detailed protocol and algorithms are described in **Text S1**.

### Statistical analysis

All linkage disequilibrium calculations (*r* ^2^) were based on 1000 Genomes Release 3 European samples. All association tests were performed using Plink v1.07. We considered *p*<5×10^−8^ to be genome-wide significant; *p*<5×10^−5^ was considered as suggestive. CD4^+^ T_EM_ abundance and proliferation correlations with age and gender were calculated by multivariate linear model implemented in R-3.0. We calculated two-sample comparisons (CD4^+^ T_EM_ cell-specific expression between genes, and H3K4me3 h/d scores between SNPs) with the Mann-Whitney test. Details of statistical analyses are described in **Text S1**.

### Data access

We make all phenotypic data (expression, peripheral abundance, and proliferation) along with eQTL results publicly available online (http://immunogenomics.hms.harvard.edu/CD4eqtl.html). Genome-wide genotype data will become available through dbGAP and through the ImmVar project. These data are potentially useful to investigators wishing to assess the potential of genetic variants in altering these molecular phenotypes.

## Supporting Information

Figure S1 Enrichment of cell-specific expression of genes within risk loci. As described in Hu et al. *AJHG* 2011, **A**) genes within risk loci of RA were the most specifically expressed in CD4^+^ T_EM_ cells (*p* = 1.00×10^−8^) followed by signal in regulatory T cells (*p* = 5.00×10^−8^). **B**) Genes within CeD were also the most strongly enriched in CD4 TEM cells (*p* = 1.43×10^−5^) followed by regulatory T cells (*p* = 3.78×10^−5^). **C**) In T1D, CD8 memory T cells showed the strongest enrichment (*p* = 2.26×10^−5^), followed by regulatory T cells (*p* = 5.13×10^−5^) and CD4^+^ T_EM_ cells (*p* = 1.29×10^−4^).
(TIFF)

Figure S2 **A**) Using a combination of magnetic and fluorescence-activated cell sorting (MACS and FACS), CD4^+^ T cells were isolated to a high degree of purity. The isolated population contained ∼97% CD3^+^ cells, ∼90% CD4^+^ cells, ∼0.4% CD8^+^ cells, and ∼0.03% CD19^+^ cells. **B**) The relative abundance (as a percentage of all sorted lymphocytes), **C**) division index (average division of all cells), and **D**) proliferation index (average division of all cells that went into division), were reproducible in 35 individuals with two blood draws at least one month apart. Pearson's *r* = 0.87, 0.57, and 0.62, respectively.
(TIFF)

Figure S3 The 46 eQTL SNPs show more overlap with H3K4me4 marks. **A**) *Cis*-eQTL SNPs were located nearer H3K4me3 peaks in CD4 T_EM_ cells than the 159 top SNPs that did not reach statistical significance at 5% FDR (*p* = 1.10×10^−7^, one-sided Mann-Whitney test). **B**) The 46 *cis*-eQTL SNPs were near larger H3K4me4 peaks (peak height) and located at smaller distances to the summit of the peaks (*p* = 9.56×10^−8^, one-sided Mann-Whitney test).
(TIFF)

Figure S4 The relative abundance of CD4 T_EM_ cells as the percentage of CD4 T cells. **A**) increased with age, at 0.11% per year; and **B**) was correlated with gender, where men on average as 2.2% more CD4 T_EM_ cells than women. The associations remained significant in a multivariate linear regression.
(TIFF)

Figure S5 SNPs associated to CeD, RA, and T1D, showed no significant association to CD4 T_EM_ cell abundance. The 119 risk alleles within densely genotyped loci showed no significant association to CD4 T_EM_ abundance as a percentage of CD4 T cells in the study by Orru et al. The shaded area shows the 95% confidence interval.
(TIFF)

Figure S6 Quantification of CD4 T_EM_ cells using X-Cyt. **A**) In each sample of enriched CD4 T lymphocytes, X-Cyt clustered all flow events based on fluorescence intensities in CD45RA, CD45RO, and CD62L simultaneously, using a seven-component multivariate Gaussian mixture-modeling. The T_EM_ cell population is shown in red, defined as CD45RA^−^, CD45RA^+^, and CD62L^low/−^. **B**) X-Cyt clustered and quantified CD4 T_EM_ cells in all samples (four random samples are shown here) in the study following the template in A). The T_EM_ cell population is shown as the red cluster in each sample. In Sample 3, the subset of the black population residing in the lower left quadrant is the light green population identified as “debris” in Panel A); they are CD62L^−^, CD45RA^−^, and CD45RO^−^.
(TIF)

Figure S7 The CFSE intensity peak present in the pooled resting wells for each subject (data underlying the green fitted curve) was modeled as a single Gaussian distribution. Its mean and variance were then used to initialize the location of the first component (undivided cells) and the variance of all components in each of the stimulated wells (data underlying the red fitted curve). The CFSE dilution peaks from stimulated wells were fitted using a one-dimensional mixture model of multiple Gaussian components of equal peak-to-peak distance and equal variance via a gradient descent optimization algorithm. A maximum of six components (five divisions) was fitted to each stimulated well; the weight of each component was allowed to be 0.
(TIF)

Table S1 Table S1 lists all genes included in the NanoString codeset, including those that did not pass quality control for downstream analyses. We list each gene's relationship to GWAS SNPs, resting and stimulated expression levels, as well as the correlation between baseline expression and post-stimulation proliferation.
(XLSX)

Table S2 Table S2 lists all densely-genotyped disease-associated SNPs for CeD, RA, and T1D included in this study. We list each SNP's association to disease(s), nearby genes included in the study, as well as association *p*-values to CD4^+^ T_EM_ relative abundance and proliferation.
(XLSX)

Table S3 Table S3 lists the 182 (densely-genotyped disease-associated) SNP-gene pairs included in the eQTL study. We list each pair's effect size and *p*-value in resting and stimulated states.
(XLSX)

Table S4 Table S4 contains all GWAS associated SNPs to CeD, RA, and/or T1D, used for designing the NanoString codeset in April 2012. For each gene, we list its lead SNP in LD, disease association (to the lead SNP), GO code, and functional description.
(XLSX)

Text S1 Text S1 includes detailed descriptions of materials, experimental methods, and statistical analyses used in our study. We provide protocols and analytical methods for 1) cell population collection, isolation, staining, stimulation, flow cytometry, and NanoString assays; 2) cell abundance and proliferation quantification; and 3) gene selection, expression analysis, and eQTL analysis.
(DOCX)
