# Supplementary figures and images for: Regulation of Gene Expression in Autoimmune Disease Loci and the Genetic Basis of Proliferation in CD4+ Effector Memory T Cells
Source: PLoS Genet. 2014 Jun 26;10(6):e1004404. doi: 10.1371/journal.pgen.1004404 (PMC4072514; doi:10.1371/journal.pgen.1004404)

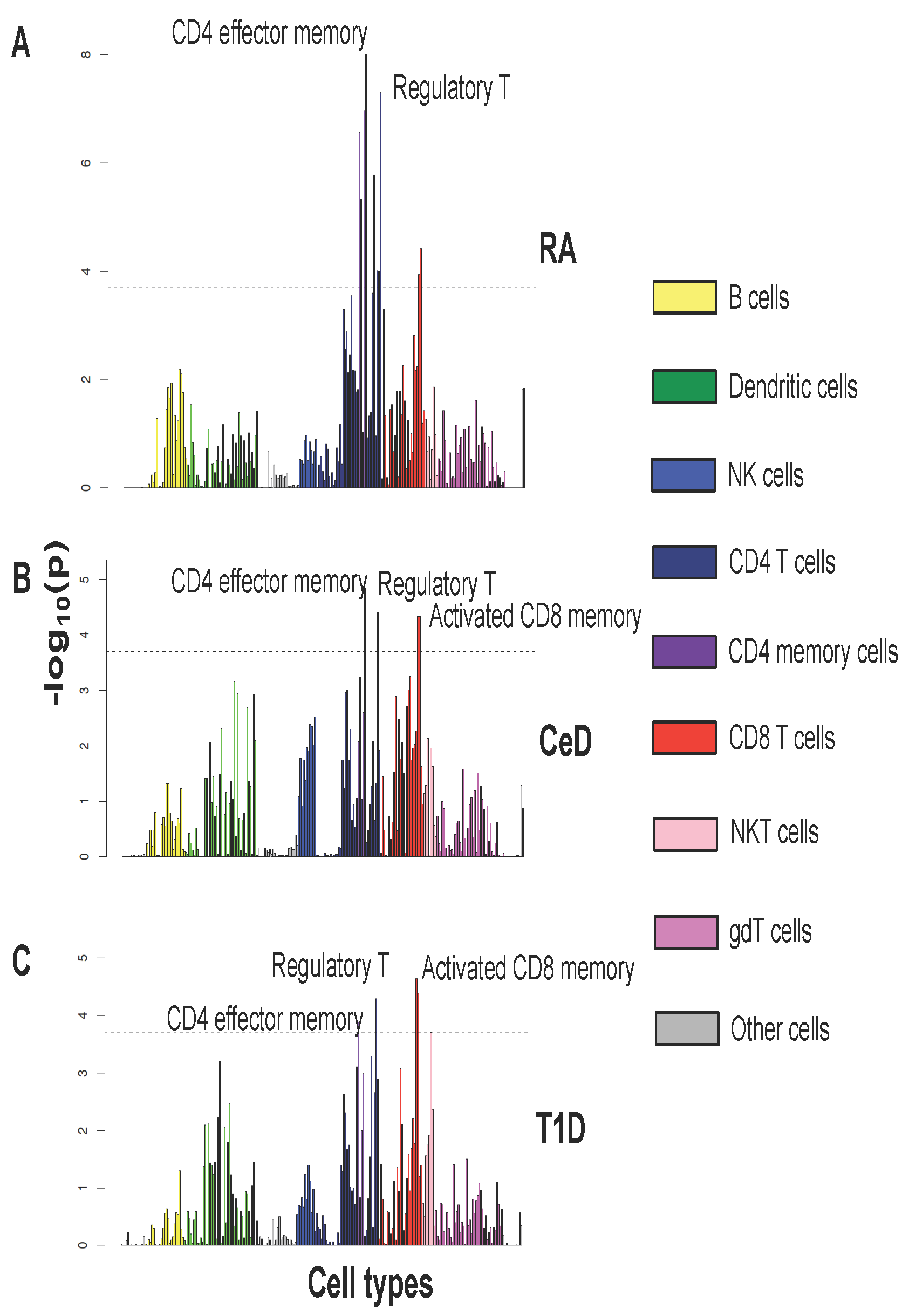

Supplement: Figure S1 — Enrichment of cell-specific expression of genes within risk loci. As described in Hu et al. AJHG 2011, A) genes within risk loci of RA were the most specifically expressed in CD4+ TEM cells (p = 1.00×10^−8^) followed by signal in regulatory T cells (p = 5.00×10^−8^). B) Genes within CeD were also the most strongly enriched in CD4 TEM cells (p = 1.43×10^−5^) followed by regulatory T cells (p = 3.78×10^−5^). C) In T1D, CD8 memory T cells showed the strongest enrichment (p = 2.26×10^−5^), followed by regulatory T cells (p = 5.13×10^−5^) and CD4+ TEM cells (p = 1.29×10^−4^). (TIFF) [file pgen.1004404.s001.tiff]

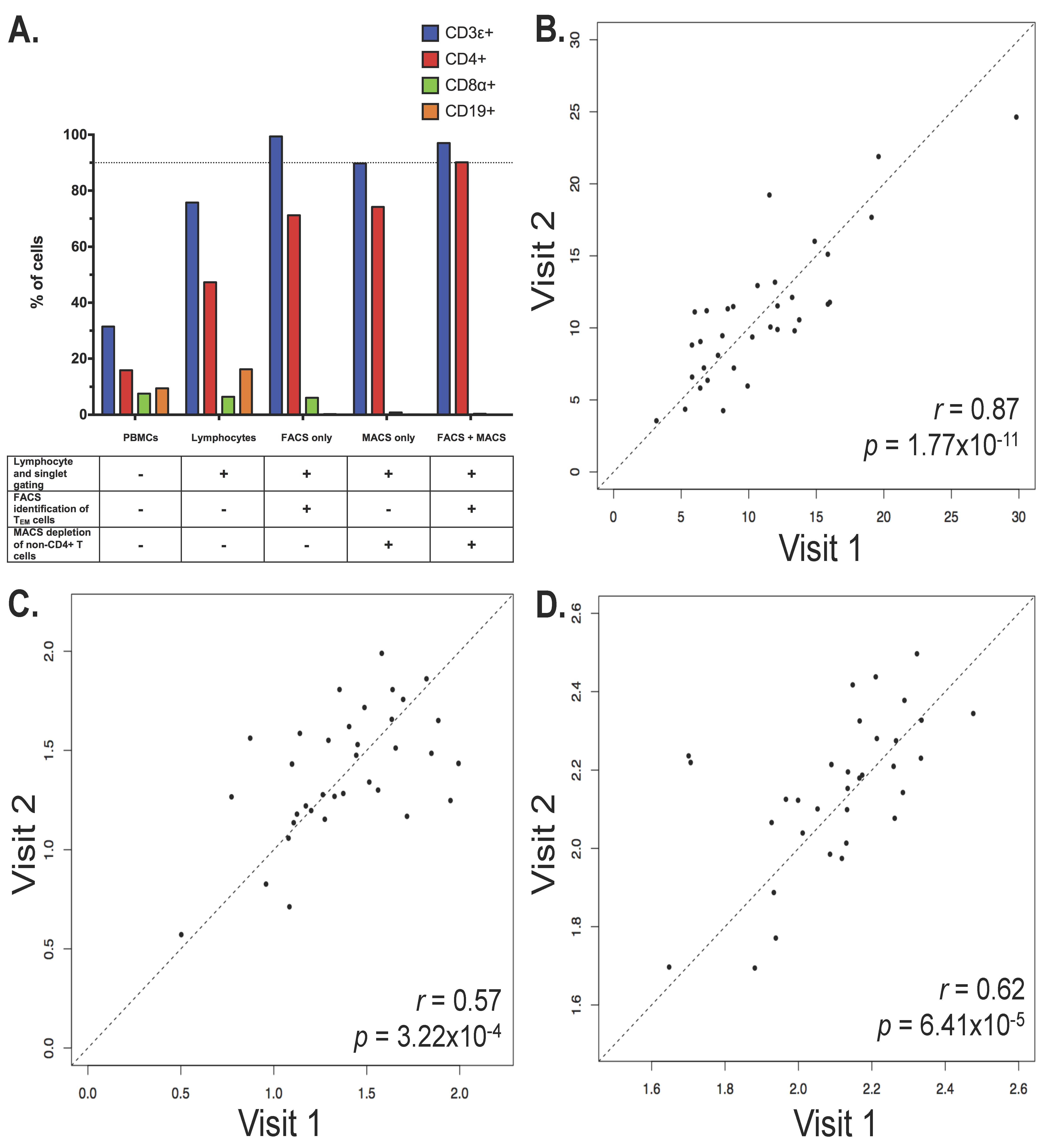

Supplement: Figure S2 — A) Using a combination of magnetic and fluorescence-activated cell sorting (MACS and FACS), CD4+ T cells were isolated to a high degree of purity. The isolated population contained ∼97% CD3+ cells, ∼90% CD4+ cells, ∼0.4% CD8+ cells, and ∼0.03% CD19+ cells. B) The relative abundance (as a percentage of all sorted lymphocytes), C) division index (average division of all cells), and D) proliferation index (average division of all cells that went into division), were reproducible in 35 individuals with two blood draws at least one month apart. Pearson's r = 0.87, 0.57, and 0.62, respectively. (TIFF) [file pgen.1004404.s002.tiff]

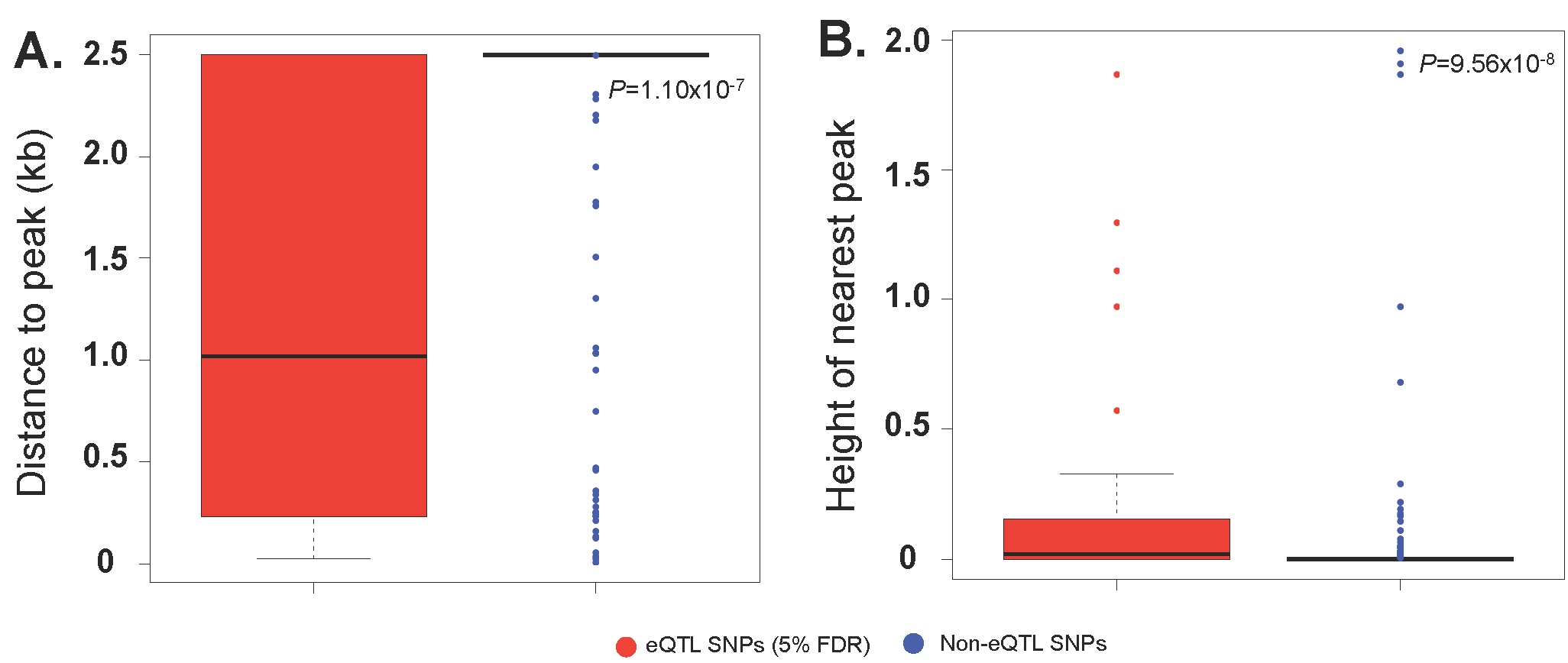

Supplement: Figure S3 — The 46 eQTL SNPs show more overlap with H3K4me4 marks. A) Cis-eQTL SNPs were located nearer H3K4me3 peaks in CD4 TEM cells than the 159 top SNPs that did not reach statistical significance at 5% FDR (p = 1.10×10^−7^, one-sided Mann-Whitney test). B) The 46 cis-eQTL SNPs were near larger H3K4me4 peaks (peak height) and located at smaller distances to the summit of the peaks (p = 9.56×10^−8^, one-sided Mann-Whitney test). (TIFF) [file pgen.1004404.s003.tiff]

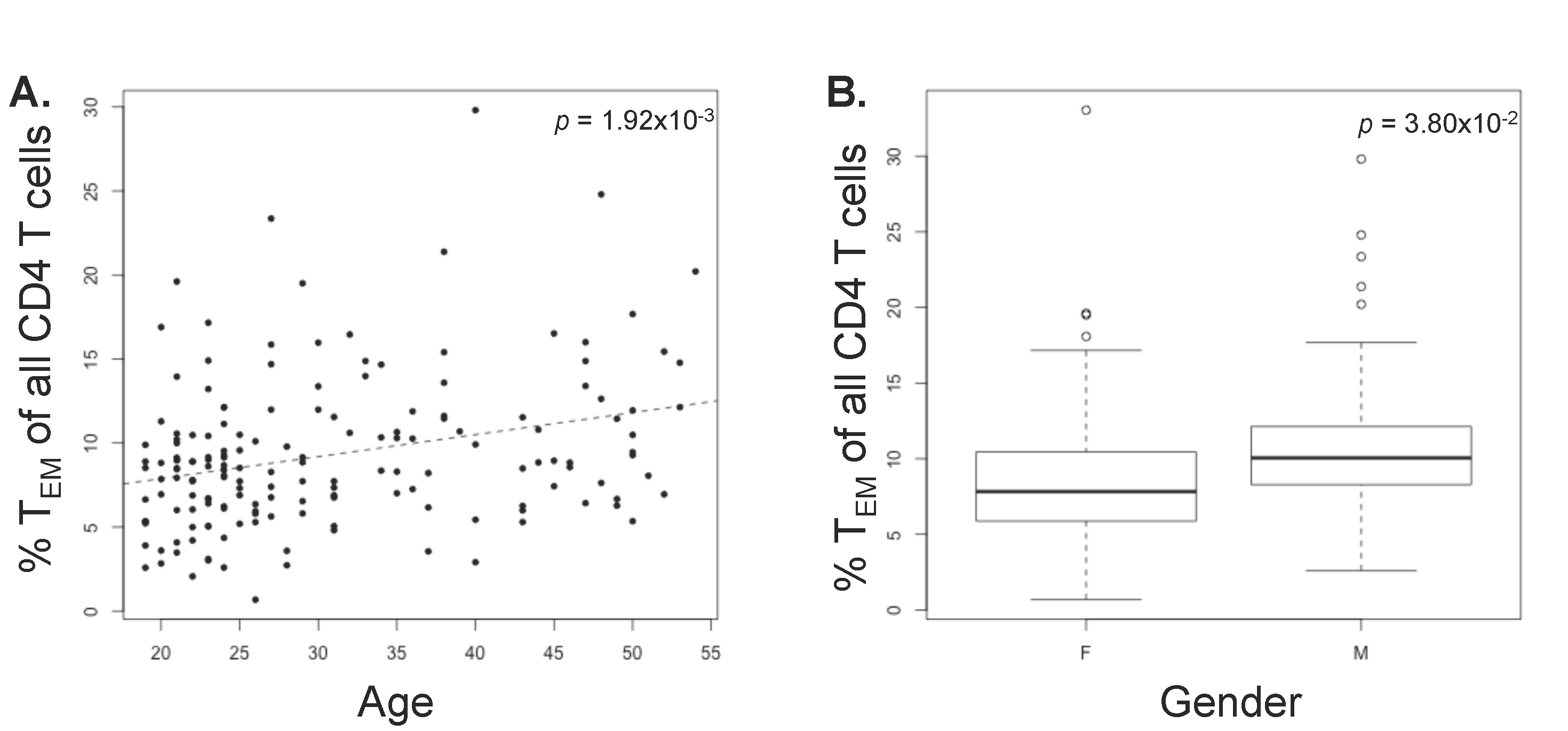

Supplement: Figure S4 — The relative abundance of CD4 TEM cells as the percentage of CD4 T cells. A) increased with age, at 0.11% per year; and B) was correlated with gender, where men on average as 2.2% more CD4 TEM cells than women. The associations remained significant in a multivariate linear regression. (TIFF) [file pgen.1004404.s004.tiff]

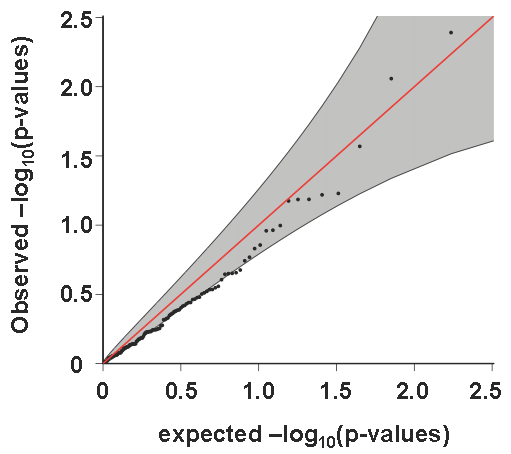

Supplement: Figure S5 — SNPs associated to CeD, RA, and T1D, showed no significant association to CD4 TEM cell abundance. The 119 risk alleles within densely genotyped loci showed no significant association to CD4 TEM abundance as a percentage of CD4 T cells in the study by Orru et al. The shaded area shows the 95% confidence interval. (TIFF) [file pgen.1004404.s005.tiff]

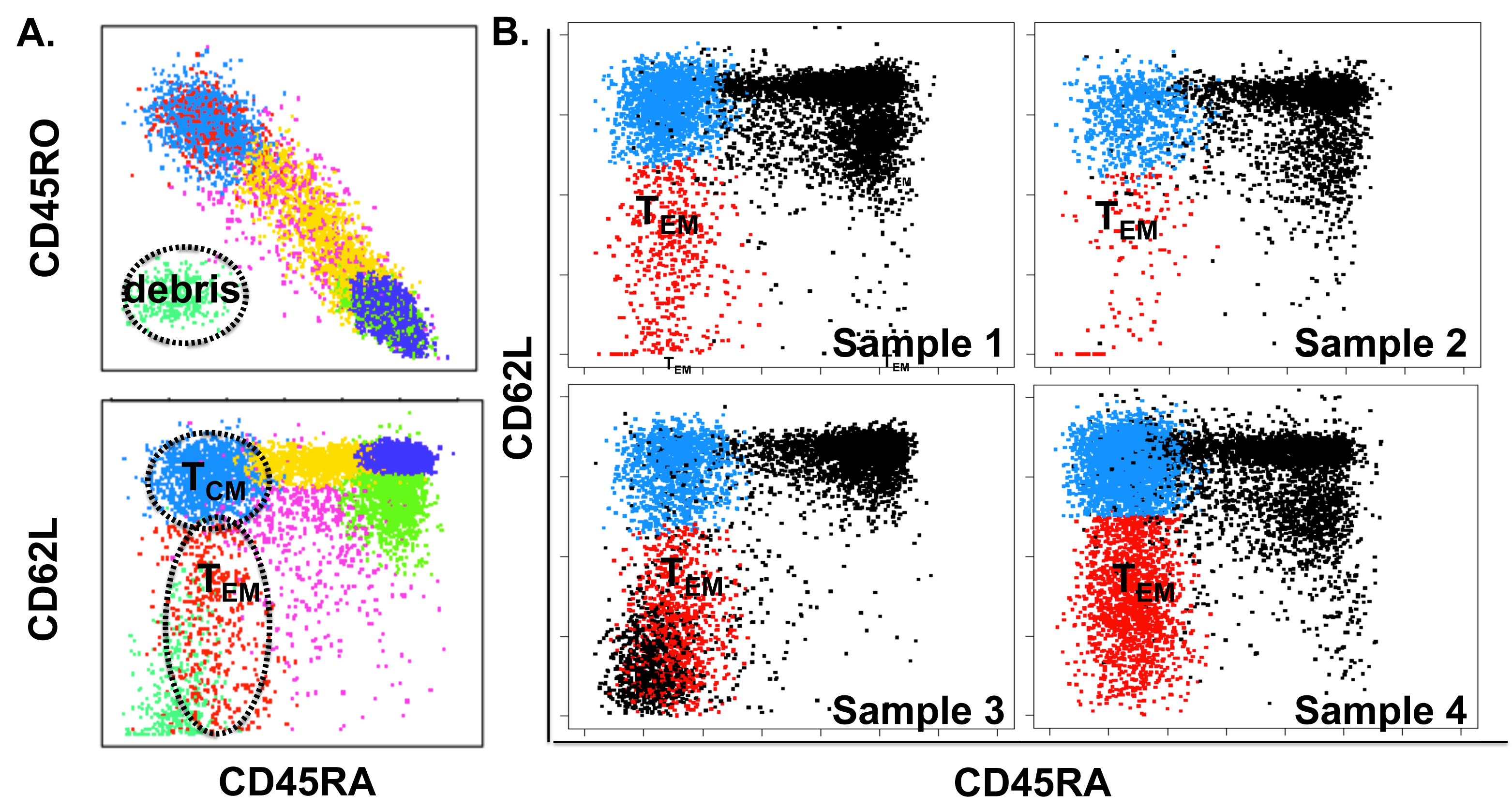

Supplement: Figure S6 — Quantification of CD4 TEM cells using X-Cyt. A) In each sample of enriched CD4 T lymphocytes, X-Cyt clustered all flow events based on fluorescence intensities in CD45RA, CD45RO, and CD62L simultaneously, using a seven-component multivariate Gaussian mixture-modeling. The TEM cell population is shown in red, defined as CD45RA−, CD45RA+, and CD62Llow/−. B) X-Cyt clustered and quantified CD4 TEM cells in all samples (four random samples are shown here) in the study following the template in A). The TEM cell population is shown as the red cluster in each sample. In Sample 3, the subset of the black population residing in the lower left quadrant is the light green population identified as “debris” in Panel A); they are CD62L−, CD45RA−, and CD45RO−. (TIF) [file pgen.1004404.s006.tif]

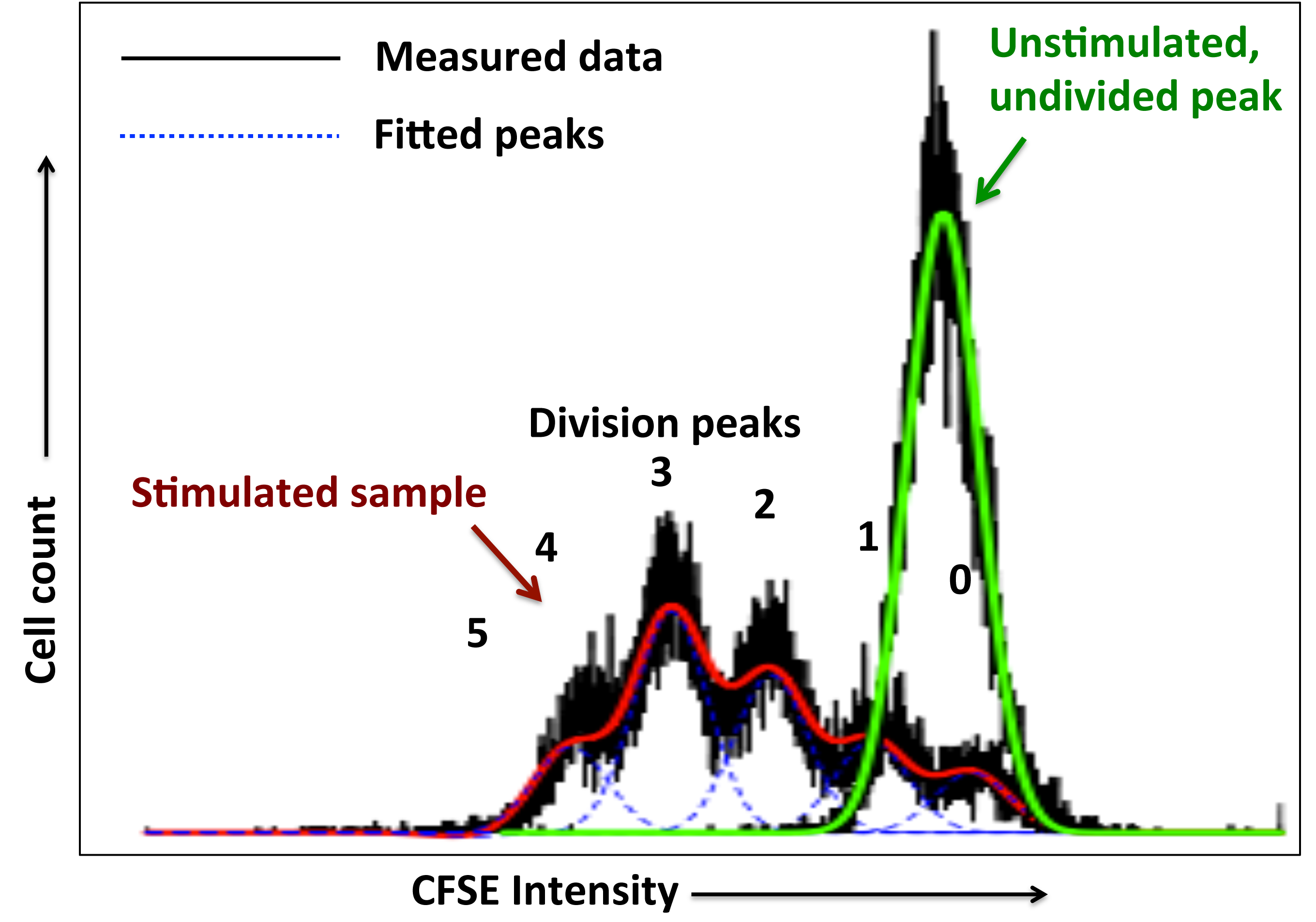

Supplement: Figure S7 — The CFSE intensity peak present in the pooled resting wells for each subject (data underlying the green fitted curve) was modeled as a single Gaussian distribution. Its mean and variance were then used to initialize the location of the first component (undivided cells) and the variance of all components in each of the stimulated wells (data underlying the red fitted curve). The CFSE dilution peaks from stimulated wells were fitted using a one-dimensional mixture model of multiple Gaussian components of equal peak-to-peak distance and equal variance via a gradient descent optimization algorithm. A maximum of six components (five divisions) was fitted to each stimulated well; the weight of each component was allowed to be 0. (TIF) [file pgen.1004404.s007.tif]
