# Supplementary material for: Regulation of Gene Expression in Autoimmune Disease Loci and the Genetic Basis of Proliferation in CD4+ Effector Memory T Cells
Source: PLoS Genet. 2014 Jun 26;10(6):e1004404. doi: 10.1371/journal.pgen.1004404 (PMC4072514; doi:10.1371/journal.pgen.1004404)
Supplement: Text S1 — includes detailed descriptions of materials, experimental methods, and statistical analyses used in our study. We provide protocols and analytical methods for 1) cell population collection, isolation, staining, stimulation, flow cytometry, and NanoString assays; 2) cell abundance and proliferation quantification; and 3) gene selection, expression analysis, and eQTL analysis. (DOCX) [file pgen.1004404.s012.docx]

Supplemental Information: Regulation of gene expression in autoimmune risk loci and the genetic basis of proliferation in CD4^+^ effector memory T cells

Xinli Hu^1-6*^, Hyun Kim^1-3*^, Towfique Raj^2,4,7^, Patrick J. Brennan^1^, Gosia Trynka^1-4^, Nikola Teslovich^1,2^, Kamil Slowikowski^1-5^, Wei-Min Chen^8^, Suna Onengut^8^, Clare Baecher-Allan^9^, Philip L. De Jager^4,7^, Stephen S. Rich^8^, Barbara E. Stranger^10-11^, Michael B. Brenner^1^, Soumya Raychaudhuri^1-4,12^

- - - 1. Division of Rheumatology, Immunology and Allergy, Department of Medicine, Brigham and Women's Hospital, Boston, MA, USA
      2. Division of Genetics, Department of Medicine, Brigham and Women's Hospital, Boston, MA, USA
      3. Partners Center for Personalized Genetic Medicine, Boston, MA, USA
      4. Program in Medical and Population Genetics, Broad Institute of MIT and Harvard, Cambridge, MA, USA
      5. Harvard Medical School, Boston, MA USA
      6. Harvard-MIT Division of Health Sciences and Technology, Boston, MA USA
      7. Program in Translational NeuroPsychiatric Genomics, Institute for the Neurosciences, Department of Neurology, Brigham and Women's Hospital, Boston, MA, USA
      8. Center for Public Health Genomics, University of Virginia, Charlottesville, VA, USA
      9. Department of Dermatology/Harvard Skin Disease Research Center, Brigham and Women’s Hospital, Boston, MA, USA
      10. Section of Genetic Medicine, University of Chicago, Chicago, IL USA
      11. Institute for Genomics and Systems Biology, University of Chicago, Chicago, IL USA
      12. Faculty of Medical and Human Sciences, University of Manchester, Manchester, UK

* These authors contributed equally to this work.

Please send correspondence to:

Soumya Raychaudhuri

77 Avenue Louis Pasteur

Harvard New Research Building, Suite 250D

Boston, Massachusetts 02446

United States of America

[*soumya@broadinstitute.org*](mailto:soumya@broadinstitute.org) 617-525-4484 (tel) 617-525-4488 (fax)

**Buffers and media**

Peripheral blood mononuclear cells (PBMCs) were washed with a cold, divalent cation-free Hycole Dulbeccos (Thermo Scientific) phosphate buffered solution (PBS). Antibody staining of CD4 T cells was performed in “fluorescence activated cell sorting (FACS) buffer”, which is PBS containing 0.5% BenchMark fetal bovine serum (Gemini Bio-Products) and 2mM EDTA (Gibco). CD4 T_EM_ cells were cultured in “basic human media”, which is RPMI 1640 media (Gibco) containing 10% Hyclone fetal bovine serum (Thermo Scientific), 5% BenchMark fetal bovine serum (Gemini Bio-Products), and supplemented with the following items and their final concentrations or volumes: 30 mM HEPES, 100 U/mL penicillin, 100 μg/mL streptomycin, 1 mM L-glutamine, 0.5 mM sodium pyruvate, 0.055 mM β-mercaptoethanol, 2.5 mL of an essential amino acid solution (Gibco), and 2.5 mL of a non-essential amino acid solution (Gibco).

**Blood collection and PBMC isolation**

For each subject, 30 mL of non-fasting blood was collected into plastic tubes spray-coated with EDTA (BD). The blood was carefully layered over Ficoll-Paque PLUS (GE Healthcare) and centrifuged at 2,000 rpm for 30 minutes to isolate PBMCs. PBMCs were washed twice with cold PBS, resuspended in FACS buffer, and filtered through a 70 μm nylon mesh. The time from blood collection to the Ficoll procedure was always less than 7 hours.

**MACS enrichment for CD4 T cells**

Magnetic activated cell sorting (MACS) was used to enrich PBMCs for CD4 T cells by depleting CD8+ T cells, monocytes, neutrophils, eosinophils, B cells, dendritic cells, NK cells, granulocytes, γ/δ T cells, and red blood cells using a CD4 T cell isolation MACS kit (Miltenyi). FACS buffer was used as the eluent.

**FACS isolation of T_EM_ cells**

FACS was used to isolate T_EM_ Cells from enriched CD4 T cells, which were labeled with phycoerythrin (PE)-conjugated anti-CD62L (eBioscience), eFluor450-conjugated anti-CD45RA (eBioscience), and allophycocyanin (APC)-conjugated anti-CD45RO antibodies (eBioscience) on ice for 40 minutes in FACS buffer. Labeled cells were then washed twice with and resuspended in FACS buffer. Cells were kept at 4°C overnight. The following morning, labeled cells were sorted on a BD FACSAria SORP flow cytometer for T_EM_ cells, which were defined as being CD45RA^-^CD45RO^high^CD62L^-/low^. T_EM_ cells were sorted into two tubes of basic human media, one for 100,000 cells and one for 120,000 cells. The first tube was used for the Nanostring gene expression assay while the second tube was used for the proliferation assay. FCS files of the sorting data were saved for automated quantification of T_EM_ cell abundance.

**T_EM_ cell stimulation**

Sorted T_EM_ cells were plated into round-bottom, 96-well plates at 20,000 cells/well. Wells for the stimulated condition received 2,000 Dynabeads coated in anti-CD3 and anti-CD28 antibodies (Invitrogen) in basic human media for a bead:cell ratio of 1:10. Wells for the resting condition received an equal volume of basic human media only. The cells for the proliferation assay and the gene expression assay were plated on separate plates. The proliferation assay plate contained two resting replicates and three stimulated replicates. The gene expression plate contained two resting replicates and two stimulated replicates. In both plates, the outer wells were avoided to minimize variability between the wells. All cells were incubated at 37°C for 72 hours.

**Proliferation assay**

Prior to plating the T_EM_ cells for the proliferation assay, cells were washed with and resuspended in room temperature PBS. They were then labeled with 0.5 μM carboxyfluorescein diacetate succinimidyl ester (CFSE; eBioscience) in room temperature PBS for two minutes. Cells were quenched with cold BenchMark fetal bovine serum (Gemini Bio-Products) and basic human media. Cells were then resuspended in basic human media and plated. Following the incubation period, cells were removed from wells and analyzed on a BD FACSCantoII flow cytometer. The two resting replicates for each sample were pooled to define the undivided cell population. FCS files of the proliferation assay were saved for downstream, automated analysis.

**Selecting target and reference genes for custom codeset**

A list of the known single nucleotide polymorphisms (SNPs) associated (*P* < 5x10^-8^) with rheumatoid arthritis, celiac disease, and type 1 diabetes, via genome-wide association and/or Immunochip studies, as of May 2011, was compiled. For each associated SNP, its implicated genomic region of interest was first defined by the furthest SNPs in linkage disequilibrium in the 3’ and 5’ directions (R^2^ > 0.5), then extended outward to the nearest recombination hotspot. All genes with any overlap with this region of interest were collected. A total of 931 unique genes were implicated by all associated SNPs. To prioritize these genes, they were annotated based on the following criteria: 1) distance to the SNP of interest; 2) Gene Ontology annotation; 3) known eQTL status; 4) a minimal expression specificity in CD4 T cells (based on mouse ImmGen data) or immune cells (based on human GNF dataset). In addition, 19 genes and ten long non-coding RNAs (lncRNAs) were added to the codeset based on immunological interest, but were not implicated by the above-mentioned association SNPs,

15 reference genes were included for the purpose of controlling for cell numbers, total RNA quantity. Due to the large metabolic demand and cytoskeleton remodeling that occurs with stimulation-induced proliferation, housekeeping genes commonly used in other molecular biology assays, such as GAPDH and actin, were not used. Instead, genes that showed stable expression levels in resting and stimulated CD4 T cells were identified in two publically available microarray datasets in Gene Expression Omnibus, GSE32607 and GSE28726, which studied primary and cloned human T cells after stimulation. Genes that showed minimal fold change in both datasets and spanned the low, medium, to high expression ranges were selected.

In total, 314 target genes and 15 reference genes were included in the custom codeset.

**Nanostring nCounter sample preparation and processing**

Cells used in the gene expression assay were not labeled with CFSE prior to plating. Following the incubation period, plates were centrifuged at 2,000 rpm for 5 minutes and the media in the wells was aspirated. Cells were lysed with 5 μL of an RLT lysis buffer (Qiagen) solution containing 1% β-mercaptoethanol. Cell lysates were stored at -80°C for 2-14 months until analysis. The standard nCounter cell lysate gene expression assay protocol was used to process the samples. All replicates were processed separately.

**Nanostring data analysis**

*Data quality control*

Raw nCounter data consisted of 343 transcriptional measurements (314 target genes, 15 reference genes, 8 negative controls, and 6 positive controls). Data was available for 265 samples (including replicates) initially with both resting and stimulated data (*n*=530). First, we identified control genes with adequate signal intensity for normalization, we required that the signal intensity of the gene exceeded double the median of negative control probe intensities in <10 samples; this resulted in 9 pre-defined control genes passing quality control. Then, we removed samples with low intensity by requiring for each sample that:

1. The mean of the natural log of the 9 control genes >2 (525/530 passing).
2. The median signal intensity of the of the 314 genes exceeded the median signal intensity of negative controls (512/530 passing).
3. The mean of the natural log of the 314 measured genes >0.5 (523/530 passing).
4. The standard deviation divided by the mean of the natural log of the 314 measured genes was >0.5 (509/530).

The resulting data set had 491 remaining samples. Finally, we applied stringent quality control to remove low intensity genes. In order to do this we required that:

1. The intensity of the gene exceeded double the median of negative probe intensities in >80% of stimulated samples (246/314).
2. The standard deviation divided by the mean of the natural log of the each gene across samples was >0.3 (292/314).
3. The standard deviation of the natural log of the each gene across samples was >1 (245/314).

The resulting data set consisted of measurements on 215 genes.

To assess if stimulated and non-stimulated samples separate naturally in expression space, as we would expect with high quality sample measurements, we calculated principal components analysis, after normalizing each gene to a mean of 0 and standard deviation of 1.

**Data normalization**

For each gene, we normalized resting and stimulated conditions together, assuming that the observed signal was the composite of a true baseline expression value, and a stimulation effect (if the sample is indeed collected from stimulated cells). Fitting the observed intensity data (*R_ij_*) in a log additive model for each gene *j* individually, allows us to determine the residuals for each individual sample *i*, *r_ij_*. Thus for each gene *i*, we fit the following model.

$$log\left( R_{i,j} \right)=\bar{X}_{j}+{I\left( {stim}_{i}=1 \right)\cdot\bar{S}}_{j}+r_{i,j}$$

where *stim_i_* represents a binary variable which is non-zero only for stimulated samples, $\bar{X}_{j}$, is the mean log expression of gene *j* across samples at baseline, $\bar{S}_{j}$ is the mean log fold change with stimulation, and *r_ij_* is the residual expression of gene *j* for sample *i.* With this formalism, the log baseline expression, log fold change with stimulation, and statistical significance for each of these parameters being >0 can be estimated with a simple linear regression model. The *r_ij_* residuals can be used to conduct association studies across individuals.

In addition to individual differences, we are cognizant residuals effects might be capturing variability in mRNA content, batch effects, reagent quality, and global shifts in expression for individual samples. In order to control for these effects, and maximize the extent to which residuals represented individual expression differences, we included additional confounder variables that might capture these effects:

$$log\left( R_{i,j} \right)=\bar{X}_{j}+{I\left( {stim}_{i}=1 \right)\cdot\bar{S}}_{j}+r_{i,j}log\left( R_{i,j} \right)+\sum\beta_{c}c_{i}$$

where *c_i_* is a series of one or more confounder variables that influences gene expression in a log linear fashion. Here the confounders that we tested as covariates in this framework included the mean of the log positive control intensities, the mean of the log control gene intensities, the chip effect (12 samples are run together), the effect of the position of the chip (both row and column), and the principal components for stimulated data. To assess the impact of each confounder variable we assessed the sum square of the residual, with the aim to use confounders to reduce the total residual across all samples and genes:

$$\sum_{i} \left( r_{i,j} \right)^{2}$$

We observed that mean of the log positive control intensities indeed captured (**Supplementary Figure X**). Briefly, we observed that the mean of the log control gene intensities (*cg_i_*) for each sample explained 52% of the sum-squared residuals – more than any other individual variable. Addition of log positive control intensities, the chip effect (12 samples are run together), or the effect of the position of the chip (both row and column) did not reduce residuals substantially beyond the reduction of *cg_i_* (<7%); we concluded that most of these effects are either minimal or captured by *cg_i_*. However, we did not that the addition of principal components did reduce residuals further. Briefly normalizing expression data for each sample to have a mean of 0 and standard deviation of 1, we calculated principal components across resting and stimulated samples separately. We observed that adding the top two components for each stimulated (*p^s^*) and non-stimulated (*p^n^*) samples explained an additional 25% of the total sum-square residual, adding additional components only improved sum squared residual explained only incrementally (<2.3% per pair of components added).

In final form we implemented the following normalization scheme:

$$log\left( R_{i,j} \right)=\bar{X}_{j}+{I\left( {stim}_{i}=1 \right)\cdot\bar{S}}_{j}+r_{i,j}+\ldots$$

$$\gamma\cdot{cg}_{i}+I\left( {stim}_{i}=1 \right)\cdot\sum_{k=1}^{2} {\pi_{k}^{s}}_{\cdot}p_{k}^{s}+I\left( {stim}_{i}=0 \right)\cdot\sum_{k=1}^{2} {\pi_{k}^{n}}_{\cdot}p_{k}^{n}$$

where γ is the linear effect for *cg_i_* and *π* is the linear effect for each of the principal component variables, *p_k_^n^*. In aggregate the use of two pairs of principal components and the log average intensity of control genes explained 77% of the sum square of residuals after linear fit.

*Assessing biological and technical reproducibility*

After obtaining residual expression values for 215 genes for each individual under resting and stimulated conditions, correlations of residuals between technical and biological replicate pairs were assessed. First, a Pearson’s r for each pair of normalized replicates was calculated. Then, technical replicates of samples collected from resting cells, technical replicates of samples collected from stimulated cells, biological replicates of samples collected from resting cells, and biological replicates of samples collected from stimulated cells were separately averaged.

To assess significance for each of these conditions, an equal number of pairs from the total pool of assayed samples, matching for stimulation status, were randomly identified. Pairs were restricted so that the data was not obtained from the same individual. For each of the four conditions, 1,000,000 sets of pairs were sampled. Significance was assessed by quantifying the number of instances the averaged correlation of randomly drawn pairs exceeded the observed averaged correlation.

**Genotyping and imputation**

Each subject was genotyped using the Illumina Infinium Human OmniExpress Exome BeadChips, which includes genome-wide genotype data as well as genotypes for rare variants from 12,000 exomes as well as common coding variants from the whole genome. In total, 951,117 SNPs were genotyped, of which 704,808 SNPs are common variants (minor allele frequency [MAF] > 0.01) and 246,229 are part of the exomes. The genotype success rate was greater than or equal to 97%. Rigorous quality control was applied that included 1) gender misidentification, 2) subject relatedness, 3) Hardy-Weinberg Equilibrium testing, 4) use concordance to infer SNP quality, 5) genotype call rate, 6) heterozygosity outlier, and 7) subject mismatches. 1,987 SNPs with a call rate < 95%, 459 SNPs with Hardy-Weinberg equilibrium *P* < 10^-6^, and 63,781 SNPs with MAF < 0.01 were excluded.

For each gene, the 500kb region (250kb to the 3’ and 5’ direction) around the transcription start site (hg19) was selected and 1000 Genomes SNPs were imputed into the genome-wide SNP data using BEAGLE Version 3.3.2. The European samples from 1,000 Genomes were used as the reference panel. Markers that had MAF < 0.05 in the reference panel as well as all indels were excluded. After imputation, markers with a BEAGLE R^2^ < 0.4 or MAF < 0.01 in the imputed samples were excluded.

***Cis*-eQTL analysis**

174 subjects had both genotyping and Nanostring expression data and were included in the eQTL analysis. Analyses were performed using R. For each gene, at rest and after simulation, each SNP within 250kb to the 3’ or 5’ direction of the transcription start site was assessed for *cis-*eQTLs using the residuals of the gene expression matrix. The imputed dosage, rather than the called minor allele number, was used to perform the linear regression. For each gene-SNP pair, a linear regression was perform, where normalized expression = β_0_ + β_1_*allelic dosage + β_2*_PC_1_ + β_3*_PC_2_ + β_4*_PC_3_ + β_5*_PC_4_ + β_6*_PC_5_ + β_7_*(factor)gender. To adjust for multiple hypothesis testing and taking into consideration the correlation among SNPs within the loci, a permutation-based *P* value for each SNP was reported. We performed 10,000 permutations per gene. In each round, the residual expression values of the samples were permuted, and the lowest *P* value achieved by any of the SNPs was recorded. The proportion of permutation *P* value smaller than the analytical *P* value was reported.

We reported the lead SNP per gene with the most significant P-value. Based on locus-wide permutation p-values of all the top SNPs, we used a cut-off of false discovery rate < 0.05, and considered those passing this threshold to be significant.

**Conditional analysis**

For each gene near a SNP within a densely genotyped locus associated to CeD, RA, or T1D, conditional analysis was performed**.** The dosage of the associated SNP (“dzSNP”) was used as a covariate, thus normalized expression = β_0_ + β_1_*allelic dosage + β_2*_PC_1_ + β_3*_PC_2_ + β_4*_PC_3_ + β_5*_PC_4_ + β_6*_PC_5_ + β_7_*(factor)gender + β_8_*dosage_dzSNP_. If more than one disease-associated SNP reside in the same gene, each SNP is conditioned on separately. We repeated the linear regression and permutations to obtain any remaining eQTL signals (FDR < 0.05) independent of the associated SNP.

**Comparison between eQTL effect sizes between resting and stimulated states**

To systematically compare the *β*_rest_ and *β*_stim_ for each gene, we used a *z*-statistic to quantify the probability that they differ (see **Table 1**). The statistic was defined as $z= \frac{\beta_{stim}-\beta_{rest}}{\sqrt[2]{{{SE}_{stim}}^{2}{{- SE}_{rest}}^{2}}}$, where *β* and *SE* are the mean and standard error of the effect size estimate from regression analysis. We than reported the *p*-value (two-tailed) assuming that *z* is distributed as standard normal.

**Enrichment of chromatin-mark overlap**

For each SNP with the strongest association to each of the 158 genes in stimulated cells, we calculated an “*h/d*” score based on the distance to and the size of nearest H3K4me3 peak to the SNP in primary CD4 memory T cells. The detailed method is described by Trynka et al. [1]. Briefly, we first identify all SNP variants in LD (R^2^ > 0.8) to the lead SNP, then locate the nearest H3K4me3 peak to any of the variants. “*H*” is the height of the peak, and “d” is the physical distance in units of base pairs to the peak. We calculated the ratio of *h/d* for each lead SNP.

**Quantification of T_EM_ cell relative abundance**

Enriched CD4 T cells labeled with antibodies against CD45RA, CD45RO, and CD62L were gated automatically in intensity space via clustering by mixture modeling, using an in-house software for large-scale cytometric data analysis [2]. Each sample was clustered using forward- (FSC) and side-scatter (SSC) to extract a purer lymphocyte population. Subsequently, a three-dimensional mixture model was fitted to each sample with 7 clusters (**Figure S6**). The CD45RA^-^CD45RO^high^CD62L^-/low^ cluster was annotated as the T_EM_ population. In a subset of samples, a small CD45RA/CD45RO/CD62L triple-negative population was identified, which was assumed to be non-lymphocytic debris and subtracted from the extracted lymphocyte population. T_EM_ abundance was calculated as the percentage of all extracted lymphocytes based on FSC/SSC (excluding any debris).

**Quantification of T_EM_ cell proliferation**

The CFSE intensity peak present in the pooled resting wells for each subject was modeled as a single Gaussian distribution. Its mean and variance were then used to initialize the location of the first component (undivided cells) and the variance of all components in the stimulated wells. The CFSE dilution peaks from stimulated wells were fitted using a one-dimensional mixture model of multiple Gaussian components of equal peak-to-peak distance and equal variance via a gradient descent optimization algorithm. A maximum of six components (five divisions) was fitted to each stimulated well. All peaks were initialized as equal in weight. The location and variance of the first (undivided) was initialized to that of the single peak of unstimulated sample. The initial distance between peaks was initialized to 250. Each iteration updated three parameters of each component (mean, variance, and mixing proportion). The algorithm converged when the residual improved by an amount less than a precision threshold (0.1% of the previous iteration) or until a maximum of 1,000 iterations was reached.

Let the number of cells in each of the *N* components (mixing proportions x total cell count) of a stimulated sample be represented by the vector {G_0_, G_1_, G_2_…G_N-1_}, where G_0_ is the number of cells that underwent zero divisions during the incubation period. Let A be the total number of cells at the start of the incubation period. Let B be the total number of divisions that all cells underwent during the incubation period. Let C be the total number of cells that underwent at least one division.

$$A=\sum_{i=0}^{N-1} {G_{i}}/{2^{i}}$$

$$B= \sum_{i=0}^{N-1} {G_{i}}/{2^{i} \times i}$$

$$C=A-G_{0}$$

Division index = B/A. Proliferation index = B/C. Since each sample was assayed in three replicates, the average proliferation and division indices of all three replicates were reported. An example of fitted division peaks is shown in **Figure S7.**

**Genome-wide association testing to CD4 T_EM_ abundance and proliferative response**

Each genotyped SNP was tested for association with each quantitative trait using linear regression. For relative CD4 T_EM_ abundance, gender (as factor), age (per year), and the top five genotypic data principal components were included as covariates. For proliferation index and division index, relative CD4 T_EM_ cell abundance and the top five genotypic data principal components were included as covariates. We considered 5x10^-8^ as the genome-wide significance threshold.

**Resting gene expression association to proliferative response**

We used a permutation-based framework to test whether individual gene transcript levels in resting CD4 Tem cells predict proliferative response. We calculated the correlation coefficient between individual residual differences for each gene, and for T cell proliferative response. In order to assess significance, we simply permuted data on T cell proliferation 10^6^ times and calculated proliferative response. The significance *p*-value is simply the proportion of instances where the absolute value of the observed correlation coefficient was exceeded by the absolute value of a coefficient resulting from permutation.

**Gene set enrichment analysis**

In order to assess whether individual genes were enriched or depleted, we compiled and curated data on gene ontology (GO) code (reference). Briefly, for each gene we assigned it a GO code if the gene or one of its homologous genes was explicitly assigned the code, or if its descendants in the GO tree [3,4]. In total this resulted in a total of 20,687 annotations. We conducted enrichment analysis in those genes passing quality control that had at least 1 annotation. To select codes for subsequent analysis, we examined only those codes were present in >5, but absent in >5 genes in our data set; this reduced the number of tested annotations to 1,008. To assess enrichment we implemented GSEA as described in Subramanian *et al*. [5], with the *p* parameter set to 0. We ordered genes by the correlation of their relative expression in resting cells with the proliferation of cells after stimulation, and assessed by permutation whether enrichment scores at any point in the ordering was statistically significant.

**References**

1. Trynka G, Sandor C, Han B, Xu H, Stranger BE, et al. (2013) Chromatin marks identify critical cell types for fine mapping complex trait variants. Nat Genet 45: 124-130.

2. Hu X, Kim H, Brennan PJ, Han B, Baecher-Allan CM, et al. (2013) Application of user-guided automated cytometric data analysis to large-scale immunoprofiling of invariant natural killer T cells. Proc Natl Acad Sci U S A 110: 19030-19035.

3. Raychaudhuri S, Altman RB (2003) A literature-based method for assessing the functional coherence of a gene group. Bioinformatics 19: 396-401.

4. Raychaudhuri S, Plenge RM, Rossin EJ, Ng AC, Purcell SM, et al. (2009) Identifying relationships among genomic disease regions: predicting genes at pathogenic SNP associations and rare deletions. PLoS Genet 5: e1000534.

5. Subramanian A, Tamayo P, Mootha VK, Mukherjee S, Ebert BL, et al. (2005) Gene set enrichment analysis: a knowledge-based approach for interpreting genome-wide expression profiles. Proc Natl Acad Sci U S A 102: 15545-15550.
